# Evaluation of some aspects in supervised cell type identification for single-cell RNA-seq: classifier, feature selection, and reference construction

**DOI:** 10.1186/s13059-021-02480-2

**Published:** 2021-09-09

**Authors:** Wenjing Ma, Kenong Su, Hao Wu

**Affiliations:** 1 Department of Computer Science, Emory University, 400 Dowman Drive, Atlanta, GA 30322 USA; 2 Department of Biostatistics and Bioinformatics, Rollins School of Public Health, Emory University, 1518 Clifton Road NE, Atlanta, GA 30322 USA

**Keywords:** Supervised cell typing, Reference dataset construction, scRNA-seq

## Abstract

**Background:**

Cell type identification is one of the most important questions in single-cell RNA sequencing (scRNA-seq) data analysis. With the accumulation of public scRNA-seq data, supervised cell type identification methods have gained increasing popularity due to better accuracy, robustness, and computational performance. Despite all the advantages, the performance of the supervised methods relies heavily on several key factors: feature selection, prediction method, and, most importantly, choice of the reference dataset.

**Results:**

In this work, we perform extensive real data analyses to systematically evaluate these strategies in supervised cell identification. We first benchmark nine classifiers along with six feature selection strategies and investigate the impact of reference data size and number of cell types in cell type prediction. Next, we focus on how discrepancies between reference and target datasets and how data preprocessing such as imputation and batch effect correction affect prediction performance. We also investigate the strategies of pooling and purifying reference data.

**Conclusions:**

Based on our analysis results, we provide guidelines for using supervised cell typing methods. We suggest combining all individuals from available datasets to construct the reference dataset and use multi-layer perceptron (MLP) as the classifier, along with *F*-test as the feature selection method. All the code used for our analysis is available on GitHub (https://github.com/marvinquiet/RefConstruction_supervisedCelltyping).

**Supplementary Information:**

The online version contains supplementary material available at 10.1186/s13059-021-02480-2.

## Background

Single-cell RNA sequencing (scRNA-seq) has revolutionized the genomics research [1–3]. Instead of measuring the average gene expression from many cells in the bulk experiments, the scRNA-seq profiles the transcriptome for each individual cell. The cell-level resolution data provide much richer information for answering a number of important questions that cannot otherwise be answered by bulk data, for example, the composition of cell types in complex tissues, the cell-to-cell heterogeneity in transcription, and the transcriptional dynamics in many biological processes such as development, differentiation, and disease progression.

There are several scientific goals in scRNA-seq studies. The first one is to decipher the cellular composition of complex tissues: one wants to know the identities of the cell types and subtypes, as well as their proportions in the tissue sample. The cellular composition itself can be of great interest in biological and clinical practices, for example, it was reported that tumor-infiltrating immune cell compositions play a vital role in understanding antitumor immune responses [4]. Once the cell types are identified, cell type–specific gene expressions are also of great interest since they enhance the understandings of cell signatures [5]. There are other goals, for example, new and rare cell type discovery [6] and pseudo-time construction to represent the temporal dynamics of transcription during a biological process [7].

For all aforementioned tasks in scRNA-seq data analyses, identifying the cell types is the first step. Thus, cell type identification for scRNA-seq is the most fundamental and critical analysis. Experimental procedure for annotating cells needs the facilitation of fluorescence-activated cell sorting (FACS) which targets specific antigens on the cell surface. Although FACS is capable of sorting several cell populations simultaneously, performing FACS is expensive which requires sophisticated instruments and meticulous experiment design [8], so it is impractical to apply it in large-scale studies. The development of computational methods for cell type identification in scRNA-seq has been a very active research field during the last several years, and many tools are currently available. These tools can be mainly categorized into two groups: unsupervised cell clustering and supervised cell typing. Here, we define the term “cell typing” as the procedure to assign cell types to cells. Earlier methods are mostly unsupervised: they cluster cells with similar profiles and then assign cell type labels according to known marker genes for each cluster [9, 10]. The development of supervised cell typing methods is more recent but has become a very active topic in the last several years [11–14]. These methods first construct a classifier from a reference dataset with known cell types. Then, for a given scRNA-seq dataset, they assign cell types for every single cell based on the trained classifier. Additionally, there also exist a few “semi-supervised” methods [15–17], where they still perform unsupervised clustering but obtain initial values of the parameters from a reference dataset.

With the growing availability of large-scale, high-quality, well-annotated scRNA-seq data, the supervised approaches have gained increasing popularity. There are several advantages of the supervised methods. First, these methods usually achieve better performance than the unsupervised ones. Secondly, they are not affected by the sample size (number of cells) of the target data, since the cell types are predicted for each cell individually. In contrast, unsupervised clustering methods require relatively larger data, and their performances depend on the number of cells. Moreover, supervised methods work better for data with imbalanced cell type proportions than the unsupervised ones, which are known to not work well when the cluster sizes are very imbalanced [18]. Moreover, the unsupervised methods often do not scale up well computationally with cell numbers [19], while the computational burden of the supervised approaches is linear to the number of cells.

In spite of all the advantages, the performance of the supervised methods relies heavily on several factors: the selection of predictive features, the construction of the prediction model, and the choice of the reference dataset. These are important questions for the investigators when they try to use supervised methods for cell type prediction in practice. For example, it is well known that discrepancies between reference and target data have adverse effects on prediction accuracy. However, such discrepancies are well expected in scRNA-seq data due to many factors such as biological/clinical conditions, sample variation, and technical artifacts (batch effects). The existing publications demonstrate their results by either directly picking a dataset [20, 21] or simply combine several datasets with batch effects removed [22]. However, a single dataset might have bias, while the combination could potentially introduce contamination by including improper reference data, which can bias the reference and hurt the prediction results. With the growing number of scRNA-seq datasets being produced, a proper suggestion on how to maximize the utility of existing datasets to construct reference datasets is in urgent need.

In the work, we perform extensive real data analyses to systematically evaluate the strategies in supervised cell typing in terms of feature selection, prediction classifier, data preprocessing, and choice of reference datasets. Although there are a few benchmark papers for comparing the performances of supervised cell typing methods [20–23], they only compare “off-the-shelf” available tools, while we take a step further to evaluate the combinations of different strategies. More importantly, we evaluate the impact of the reference data and potential strategies for processing the reference data, which have never been investigated before to the best of our knowledge. Based on our analyses, we provide a guideline and rule of thumb for using the supervised cell typing methods.

## Results

### Study design

#### Methods under comparison

We include the following nine supervised cell typing methods in the comparison, which cover a wide range of different strategies for supervised cell typing:

- Three off-the-shelf supervised learning methods: random forest [24], SVM with linear kernel, and SVM with radial basis function kernel [24]
- Two supervised cell typing methods specifically designed for scRNA-seq data based on the correlation between the target and reference data: scmap [11] and CHETAH [13]
- Two supervised deep learning methods: multi-layer perceptron (MLP) [25] and graph-embedded deep neural network (GEDFN) [26]
- Two semi-supervised deep learning method: ItClust [16] with transfer-learning and MARS with meta-learning concepts [17]

There are several other supervised cell typing methods available for scRNA-seq. For example, scSorter [27] borrows information from lowly expressed marker genes to assign cells; scPred [12] adopts a principal component analysis (PCA)-based feature selection; SingleCellNet [28] uses top-pair transformation on gene space and selects informative paired genes as features; CellAssign [29] builds a probabilistic model with some prior knowledge of cell markers, etc. But according to a recent comparison [20], SVM with rejection, scmap, and CHEAH are among the best performers, so we decide not to include more such methods. GEDFN is a method designed for predicting phenotype from bulk expression but can be directly applied to scRNA-seq cell typing. We include it because we want to understand whether incorporating gene network information can improve the results. ItClust is a semi-supervised method which only uses the reference data to obtain initial values for unsupervised clustering in target data. MARS uses a meta-learning concept to construct cell type landmarks by jointly embedding both annotated and unannotated data without removing the batch effects and then assigns cell types based on the learned embedding space. We want to evaluate the performances of these semi-supervised methods under different scenarios.

#### Feature selection methods

It is known that feature selection plays an important role in many high-throughput data analyses, including scRNA-seq cell clustering and supervised cell typing. Since most genes are not cell type specific, including them in the prediction model will dilute the signal and impair the prediction accuracy. Most cell typing methods have a feature selection step. When one evaluates the performance of a method, it is unclear whether the performance gain/loss comes from the feature selection or the method itself. We want to merely investigate the impact of feature selection, so we decouple this step from the prediction. We include two unsupervised feature selection methods: one is Seurat V2.0 [10], which is based on marginal gene expression and variation, and the other is FEAST [30], which is based on unsupervised consensus clustering followed by *F*-test for ranking features. Briefly speaking, FEAST first performs unsupervised consensus clustering (similar to that in SC3) and then performs *F*-test on the clusters to calculate the feature significance and rank features. We also include one supervised method using *F*-test to select features from the reference dataset where the cell types are known.

Another aspect of the problem is whether to select features from the reference or target datasets. It is obviously more desirable to select features from the reference data, since one only needs to perform feature selection and prediction model construction once for each reference dataset. On the other hand, selecting features from the target data might be able to capture the target data characteristics more accurately and improve the prediction accuracy. In fact, ItClust suggests selecting features from the target data. In such a case, re-training the prediction model for each target data might be worth the extra computational burden. Thus, we evaluate the Seurat and FEAST feature selection in both reference and target datasets. In the meantime, we also investigate the impact of feature number and decide to pick the top 1000 features for downstream analysis (Additional file 1: Section S5) in all feature selection procedures. As a baseline, we also include results from not selecting features at all. Altogether, we test 6 feature selection procedures.

#### Datasets

All datasets used in this study are listed in Additional file 1: Tables S1-S3. Briefly, we include multiple datasets from human peripheral blood mononuclear cells (PBMC), human pancreas, and mouse brain. For human PBMC datasets, we include studies from lupus patients [31] using 10X Chromium (denoted as “Human PBMC lupus”) and frozen (pbmc1) and fresh (pbmc2) samples [32] processed by three protocols including 10X Chromium, Smart-seq2, and CEL-seq2. For human pancreas datasets, we include three human pancreas datasets [33–35]. In mouse brains, the cell type composition is more complex and has variations among the brain regions. To simplify, we focus on the frontal cortex and hippocampus regions from adult mouse whole brain study [36] using Drop-seq (denoted as “Mouse brain FC” and “Mouse brain HC”), prefrontal cortex region from adolescence and addiction study [37] using 10X Chromium (denoted as “Mouse brain pFC”), cortex samples from [32] processed by DroNc-seq (denoted as “Mouse brain cortex”), and samples with frontal cortex regions extracted from [38] processed by 10X Chromium (denoted as “Mouse brain Allen”). The cell types from the above datasets are annotated in the literatures by unsupervised clustering and known marker gene expression. To ascertain the computationally derived annotations do not bias toward certain computational prediction methods, we also include a human PBMC datasets with 10 cell subpopulations from a healthy donor, where the cell types were identified by FACS sorting [39].

The chosen datasets enable us to investigate different scenarios in terms of reference data selection. We conduct many tests with different scenarios for the reference and target data discrepancies, including the following:

- Individual difference: when reference and target data are from different individuals. In this case, the discrepancy only comes from biological variations.
- Condition difference: when reference and target data are from different conditions, including protocol difference (10X Chromium vs. Smart-Seq2), sample collection difference (e.g., frozen and fresh tissues), lab effect (data generated by different laboratories), biological difference (e.g., different brain regions), and clinical difference (e.g., different disease status). These tests cover a wide range of biological, clinical, and technical discrepancies between reference and target datasets.

In addition to the discrepancies between reference and target, we also investigate the strategy of using a “pooled” reference: to combine data from many individuals with the same or different conditions together. Such a strategy can increase the reference data size and potentially average out the individual or condition variation (“pooling effect”). In order to distinguish whether the performance gain/loss comes from the increased reference data size or the pooling effect, we also perform down-sampling on the reference data to make a fair comparison. Meanwhile, we are also curious about whether purifying the reference dataset can improve prediction performance. We adopt two strategies to remove “noisy cells” (cells that are not tightly clustered) in the reference and investigate the prediction performance with the purified reference.

#### Evaluation metrics

We use three metrics to evaluate the prediction results:

- Accuracy (Acc), which is the proportion of correct cell type assignments among all cells, directly evaluates the overall final cell typing accuracy.
- Adjusted Rand Index (ARI), which evaluates the clustering similarity between ground truth and prediction, without considering the accuracy of the assignment of cell types for clusters.
- Macro F1, which is a harmonized factor weighing precision and recall rate while considering all classes having equal contributions. It is a suitable metric when the cell type proportions are highly imbalanced.

In addition, we also benchmark the computational performance of all methods.

#### Summary of the study design

We evaluate all the combinations of the aforementioned factors in the supervised cell typing: different prediction methods, feature selection methods, and choices of reference data. Overall, we obtain results for 29 predictions (Additional file 2: *Results_Summary_Table.xlsx*), 6 feature selection, 9 prediction methods, and 4 metrics (including running time as an additional metric), which produce a total of over 5000 results. All results are provided in an R data frame in Additional file 3: *experiment_results.RDS*.

### *F*-test on reference datasets along with MLP achieves the best overall performance

We first evaluate the overall impact of different feature selection and prediction methods across all experiments. Since each experiment has a different baseline performance, i.e., the prediction accuracies are higher in some experiments than others, we remove such baselines to compute the performance gains or losses merely induced by feature selection and prediction methods. By doing so, the results from all experiments can be summarized altogether. More details about the procedure are provided in Additional file 1: section S1.

**Fig. 1:**
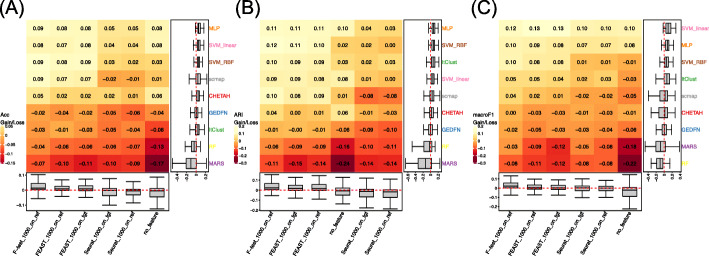
Prediction performance gains/losses with different combinations of classifiers and feature selection strategies on all experiments. **A** Accuracy. **B** ARI. **C** Macro F1. The performance gains/losses for all combinations are illustrated by the heatmap. The heatmaps are sorted by the average values of the rows and the columns, and thus, the entry at the top left corner represents the most performance gain combination. The boxplots on the right and bottom sides illustrate the marginal performance gains/losses from classifiers and feature selection methods. The red dotted lines in the boxplots are reference lines at 0 (no gain nor loss)

We summarize the performance gains/losses of all combinations of feature selection methods and classifiers in Fig. 1. The heatmap shows the results for the combinations, and the boxplots on the sides show the marginal gains/losses from each feature selection and classifier alone. The heatmaps are sorted by the average values of the rows and the columns, so that the entry in the top left corner represents the best overall performer. For example, the vertical boxplots in Fig. 1A show that the median gain in the accuracy of using MLP as the predictor is 0.053, and the horizontal boxplots in Fig. 1A show that the median gain for using *F*-test on reference data to selection feature is 0.013. The heatmap shows that combining *F*-test on reference and MLP, which is the best combination, provides a gain of accuracy of 0.09. Overall, we observe that using *F*-test on reference data as a feature selection method is the best, whereas using Seurat on reference data, Seurat on target data, and no feature selection are among the worst performers. The results also reveal that FEAST produces better feature selection than Seurat not only in unsupervised clustering tasks [30] but also in supervised cell typing. In terms of classifiers, MLP is the best overall, but SVM with both linear and RBF kernels provides comparable results. These results are consistent with the ones reported in [20], where the SVM with rejection has the best performance. These conclusions in general hold for other metrics (ARI and Macro F1), only that the SVM with linear kernel has a slight edge over MLP in Macro F1. Among the two semi-supervised methods, ItClust performs reasonably well and ranks the 3rd when using ARI as measurement, only slightly behind MLP and SVM. MARS has poor performances based on our tests: it ranks the last on average accuracy and ARI, and the results are highly variable, indicating poor robustness.

### Impact of the reference data size

After investigating all experiments together, we wonder if some properties of the reference dataset may affect the combination of feature selection strategies and prediction methods. We start by investigating the reference data size, since it has long been recognized as an important factor in supervised learning.

**Fig. 2:**
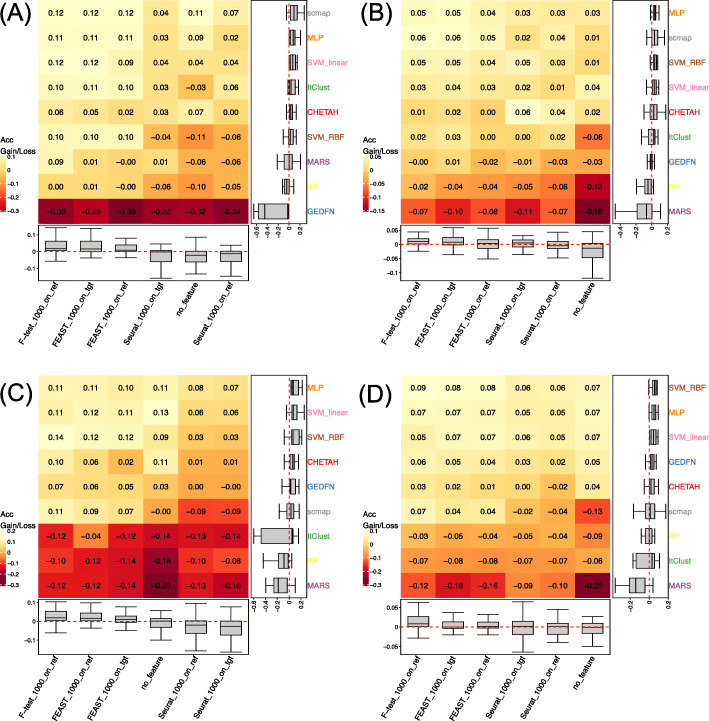
Prediction accuracy gains/losses with different reference sizes. **A** 0–1000 cells. **B** 1000–5000 cells. **C** 5000–10,000 cells. **D** 10,000+ cells. scmap ranks 1st in **A** and 2nd in **B**, but when cell number increases, scmap ranks 6th both in **C** and **D**. MLP ranks steadily as 1st or 2nd. SVM with RBF kernel first ranks 6th in **A** but later ranks 1st in **D**. The *F*-test on reference data ranks steadily as 1st among all feature selection methods

First, we categorize the sample size of all experiments into four bins: (1) 0 to 1000 cells, (2) 1000 to 5000 cells, (3) 5000 to 10000 cells, and (4) 10,000 cells and higher. We clearly observe that when the training size is small (< 1,000 cells), scmap along with three feature selections, including FEAST on target, *F*-test on reference, and FEAST on reference, all achieve an accuracy gain of 0.12 (Fig. 2A). However, when cell number increases, the accuracy gain of scmap gradually drops comparing to other methods. This is not because scmap becomes worse, rather, it is because the machine learning and supervised deep learning methods become better for larger reference data. One can clearly see that when reference data has 5000 to 10,000 cells, scmap still ranks the second (Fig. 2B), but with even larger reference data, scmap is among the worst performers (Fig. 2C, D). We observe a similar pattern for ItClust and MARS, i.e., they rank better when cell numbers are smaller. GEDFN is on the other direction: it performs very poorly when there are less than 1000 cells in the reference data but becomes much better with larger reference data. Overall, MLP and SVM consistently perform well in all four categories, especially when the reference data is large (> 5000 cells). Similar patterns have been observed in ARI and Macro F1 as shown in Additional file 1: Figure S1. These results indicate that for small reference data, it is better to use scmap, while one should switch to MLP or SVM for large reference data. As for the feature selection method, we observe *F*-test on reference steadily generates the best result.

### Impact of number of cell types

In addition to sample size, the number of classes is another critical factor influencing classification accuracy. In cell typing, there are often a few (< 10) major cell types in one tissue and many subtypes under each major type. The sub-cell types tend to have similar gene expression profiles, which makes them difficult to be distinguished. As a consequence, performances of feature selection and classifiers may vary.

**Fig. 3:**
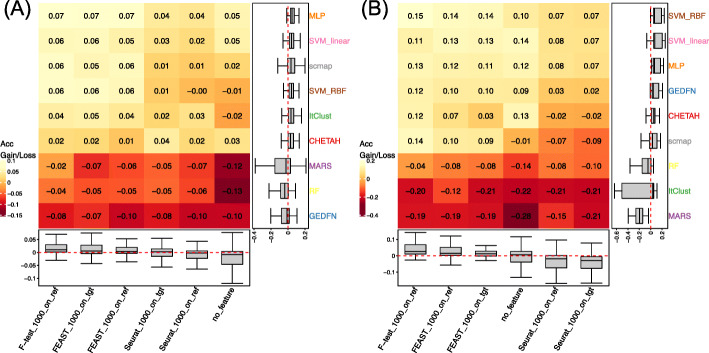
Prediction accuracy gains/losses with different numbers of cell types in the reference dataset. **A** ≤ 10 cell types. **B** > 10 cell types. scmap ranks 3rd when a smaller number of cell types but ranks 6th when there are more cell types. SVM with RBF kernel first ranks 4th and then 1st when the number of cell types increases. The *F*-test on reference data still ranks steadily as 1st among all feature selection methods

We split the experiments into two categories by the numbers of cell types, using 10 as a threshold. The observations show when cell type number is less than 10, MLP performs the best with an accuracy gain of around 0.07 (Fig. 3A). CHETAH and ItClust have an average performance. However, when the number of cell types increases to larger than 10 (Fig. 3B), scmap becomes comparatively worse than machine learning and deep learning methods because correlation-based methods cannot fully identify the differences between sub-cell types, while MLP and SVM can better capture the nonlinear relationship thus provide better predictions. The performance of MARS also drops because it has difficulties discriminating sub-cell types when assigning cell types with similar cell type landmarks. As for feature selection, the *F*-test on the reference dataset is still a better option. Similar patterns have been observed in ARI and Macro F1 as shown in Additional file 1: Figure S2.

### Impact of cell type annotations

For all the above analyses, the cell types of both reference and target data were computationally identified. They first conduct unsupervised cell clustering based on transcriptomic profiles and then annotate the cell types according to known marker gene expression. These data are not exactly “gold standard” since there could be mislabels in the annotation. In order to make sure our previous results are not biased by the computational cell type annotation procedure, we perform additional analyses using data where the cell types are experimentally identified using FACS cell sorting. Note that there are only a handful of datasets with cell types identified by experimental cell sorting. Moreover, since the cell surface markers are not available for all cell types, these data are often biased toward certain cell types. Thus, these data cannot be used as a reference and can only be used as a target in method evaluation.

We conduct four additional analyses with “Human PBMC FACS” as the target dataset (Additional file 1: Section S2). Our result again shows that MLP and SVM have overall better performances compared to other prediction methods, and the *F*-test feature selection on the reference dataset has higher performance gains with less variations (Additional file 1: Figure S3). These results show that our previous conclusions are not biased by computationally derived annotations.

### Impact of data preprocessing

To alleviate the noises in scRNA-seq data, a number of methods have been developed for scRNA-seq data preprocessing, including batch effect removal and missing data imputation. We perform a series of analyses to evaluate whether the preprocessing helps supervised cell type identification.

We first evaluate the impact of missing data imputation. In a recent study [40], several imputation methods were evaluated to assess the accuracy and the usability of downstream analysis. We choose three outperforming methods MAGIC (smooth-based) [41], SAVER (model-based) [42], and scVI (data reconstruction based on deep learning) [43] to impute both reference and target datasets and then train a classifier for cell type prediction. Since we observe that the MLP classifier with *F*-test feature selection produces the best prediction, we only evaluate the impact of imputation on this combination. Our results (Additional file 1: Figure S4) indicate that no imputation method steadily outperforms the one without imputation under all scenarios. Thus, we believe that imputation may not be a necessary preprocessing step for supervised cell typing.

We next evaluate the impact of batch effect removal. There are several methods specifically designed for scRNA-seq to remove the batch effect, and they are comprehensively compared in [44]. Here, we apply two popular batch effect removal methods: Harmony [45] and fastMNN [46, 47] on the data, and again compare the prediction performance to the original ones without removal. The same as in imputation, we only perform such comparison on the MLP with *F*-test combination. Our results (Additional file 1: Figure S5) show that there are no significant differences with or without removing the batch effect. In fact, the ones without batch effect removal have slightly better performances in most cases. Therefore, we conclude that batch effect does not affect the prediction performance and the correction may not be required, and we directly concatenate the datasets to perform the following analyses.

### Condition effect

Next, we want to know how the difference between the reference and target datasets will affect the prediction. As stated in the previous Study Design: “Datasets” section, we categorize the discrepancies between the reference and target datasets into individual effect and condition effect. In our definition, the individual effect describes individuals from the same dataset under the same technical and clinical conditions, so the difference between reference and target data only comes from biological variations. The condition effect is broader, including technical artifacts such as batch effect as well as other biological and clinical condition differences. Thus, the impact from the individual effect should be considerably smaller than the condition effect. In our design, we use individual effect as baseline and benchmark different types of condition effects toward it. Within this section, we only present the results from using *F*-test on the reference dataset for feature selection and using MLP as the classifier, since they are proved to have the best results in previous sections.

#### Comparing individual effect, region effect, and dataset effect in mouse brain data

We first investigate the condition effect in mouse brain data. We fix the target dataset as one mouse subject from “Mouse brain FC.” For individual effects, we use each of the other six mouse subjects within the same brain region from the same dataset as a reference. To evaluate the biological effect, specifically the brain region effect in this case, we use each of the six subjects within “Mouse brain HC” from the same dataset as a reference. For dataset effect, we use mouse subjects in the other two datasets (“Mouse brain pFC” and “Mouse brain cortex”) as a reference. For fair comparisons, we only predict the major cell types in mouse brain datasets. More details of the dataset description are provided in Additional file 1: Section S2.

**Fig. 4:**
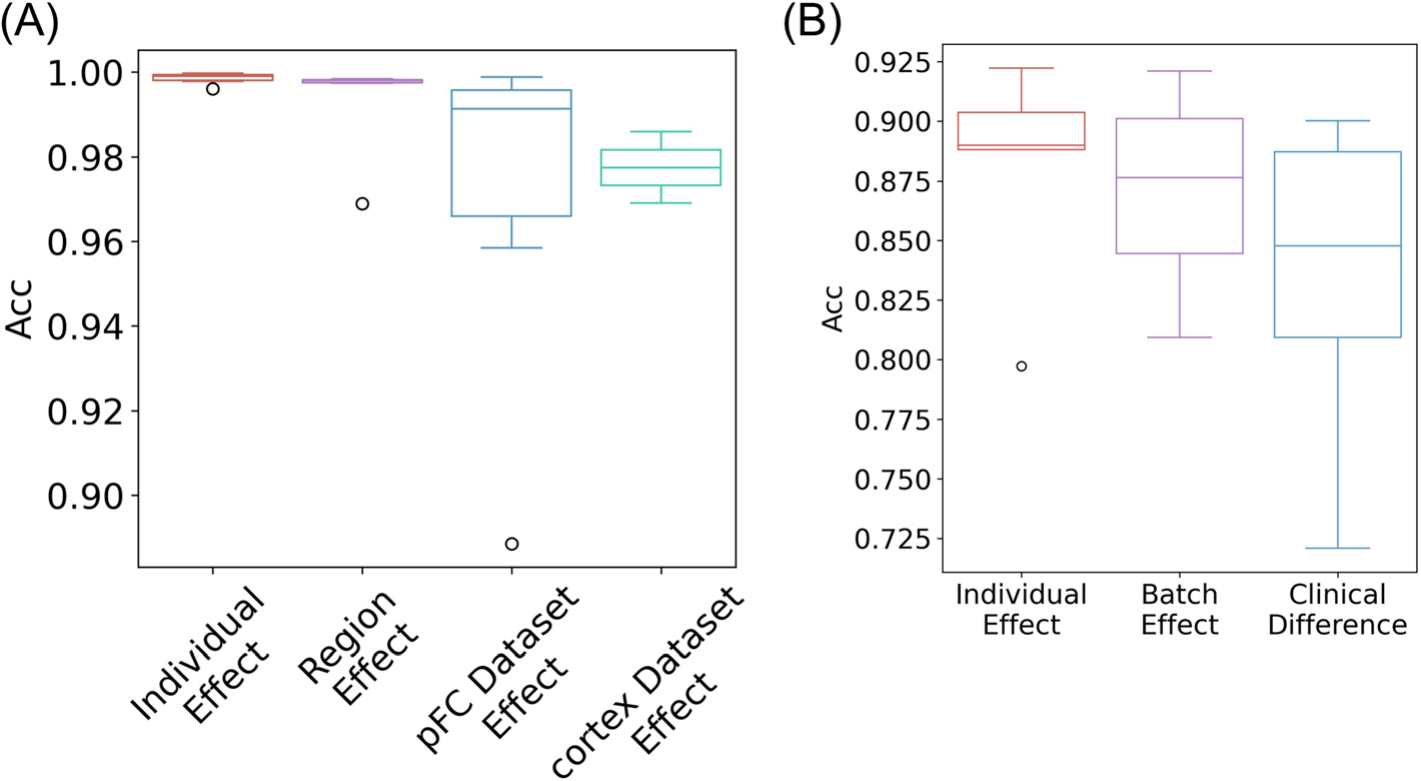
Impact of discrepancies between reference and target dataset. **A** Individual effect, region effect, and two dataset effects from the mouse brain datasets. The target is set as a mouse object from “Mouse brain FC”. The individual effect box contains 6 results from “Mouse brain FC”. Region effect uses subjects from “Mouse brain HC” to predict the target, which contains 6 results. “Mouse brain pFC” dataset effect contains 6 mouse subjects with saline treatment. “Mouse brain cortex” dataset effect contains 2 cortex samples. **B** Individual effect, batch effect, and clinical difference from the “Human PBMC lupus” dataset. The individual effect box contains 7 results from batch 1. The batch effect box contains individuals from batch 2 but under the same condition to predict the target which contains 8 results. The clinical difference contains individuals from batch 2 but under IFN-β treatment to predict the target which contains 8 results

We obtain four sets of results in total: individual effect, region effect, and two dataset effects. The results are summarized in Fig. 4A. Overall, these results show that the individual effect is very small, i.e., it achieves the mean accuracy and ARI at almost 1 and Macro F1 high as 0.961 (Fig. 4A; Additional file 1: Figure S6A, B). In the meantime, the variance of prediction results using different subjects as reference is also very small. The region effect is slightly stronger, i.e., it causes 0.5% less mean accuracy and 0.4% less mean ARI compared to the individual effect, while the mean macroF1 decreases down to 0.915. The dataset effect is the strongest. When using “Mouse brain pFC” as a reference (which contains 6 mouse subjects), the performance decreases rapidly along with a large variance. The mean of accuracy drops to 0.971, ARI to 0.947, and Macro F1 to 0.892. When using “Mouse brain cortex” as a reference (which contains two cortex samples), the mean accuracy is 0.977, the mean ARI is 0.936, and the mean Macro F1 is 0.918. These results are as expected, since the dataset effect contains both biological and technical discrepancies.

#### Comparing batch effect and clinical difference in Human PBMC

Another common condition effect is clinical difference, and we evaluate its impact on predictions using “Human PBMC lupus” data. We choose one of the eight lupus patients in batch 1 as a target. For baseline individual effect, we use the other seven individuals from the same batch to predict the target as the baseline. For batch effect, we use the other eight lupus individuals from batch 2 under control status to predict the target. For clinical difference, we use the same eight individuals from batch 2 with IFN-β treatment to predict the target again. The results are summarized in Fig. 4B and Additional file 1: Figure S6C, D.

Again, we observe that the individual effect is the smallest with a mean accuracy of around 0.885, mean ARI of 0.786, and mean Macro F1 of 0.762. The reason why the overall performance of predicting human PBMC cell types is lower than those in mouse brain is because there exist similar sub-cell types in the dataset, i.e., CD4+ T cells and CD8+ T cells; thus, the signal in the data is weaker than the mouse brain data. Compared to individual effect, the pure batch effect achieves a slightly worse performance with 0.872 in mean accuracy, 0.779 in mean ARI, and 0.717 in mean Macro F1. As for the clinical difference, the prediction becomes worse with 0.838 in the mean accuracy, 0.724 in the mean ARI, and 0.673 in the mean Macro F1.

#### Conclusions on conditional effects between reference and target datasets

The results from mouse brain and human PBMC datasets reveal several important points. First, the individual effects (caused by biological variance) are small, evidenced by the best performance from using subjects from the same dataset under the same condition as a reference. Secondly, the biological effect is also not significant for predicting major cell types, e.g., using the hippocampus from the same dataset as a reference can accurately predict major cell types in the frontal cortex. This indicates the similarities in the gene expression profiles of major cell types between the frontal cortex and hippocampus and that the major cell type differences are much stronger than the brain region differences. However, despite individual effect and region effect both achieve high performances, these two cases are impractical in real data scenarios since most of the prediction will happen across datasets in practice. When predicting across datasets, the performance becomes worse. This is reasonable since the dataset effect contains both technical and biological/clinical effects. However, our results indicate that the performance reduction in predicting major cell types across datasets is not severe: the accuracy only drops by less than 0.02 in both datasets. When reference and target data have significant clinical differences, there will be some but not dramatic performance reductions (Fig. 4B; Additional file 1: Figure S6C, D). In general, we conclude that the dataset difference, when there is no strong clinical difference, does not have a significant impact on predicting major cell types. With clinical differences, one should expect some performance reductions. However, in those cases when investigators cannot find the reference data with a matching clinical condition, using data from normal control as the reference is not a terribly bad idea.

### Pooling references improves the prediction results

After obtaining a better understanding of how the discrepancies between reference and target datasets affect the prediction, a natural thought is to combine reference datasets to reduce bias. To validate this, we perform reference dataset “pooling” to investigate whether it can improve the prediction. We fix the target dataset as one subject and pool data from multiple individuals and different conditions to create a larger reference for prediction. In order to understand whether the prediction improvement is from the increased reference data size or data pooling effect, we also down-sample the pooled reference to eliminate the reference size effect. We choose the results from individual effects as baselines in these comparisons. Reference pooling is conducted under both intra-dataset and inter-dataset scenarios. We choose individuals or subjects from “Human PBMC lupus,” “Mouse brain FC” with major cell types, and “Mouse brain FC” with sub-cell types to perform intra-dataset prediction. For inter-dataset prediction, we use mice from “Mouse brain FC” to predict mice in “Mouse brain pFC.” More details about the dataset selection and processing are provided in Additional file 1: Section S2.

#### Individual effect, pooling effect, and downsampled pooling effect

**Fig. 5:**
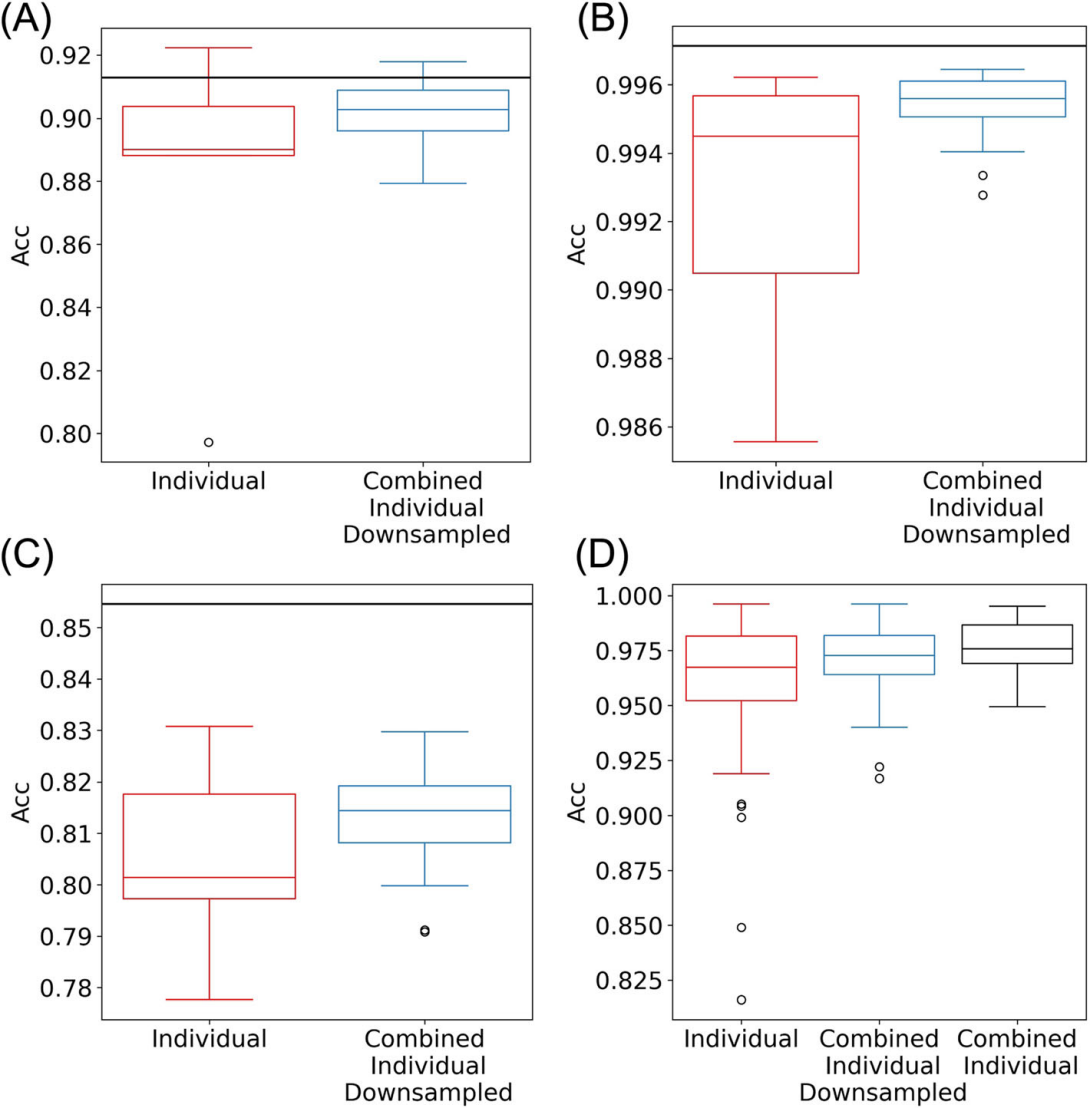
Impact of “pooling” on individual effect under intra-dataset and inter-dataset scenarios. Accuracy comparisons among individual effect (red box), down-sampling strategy (blue box), and “pooling” all individuals (black line for intra-dataset, black box for inter-dataset). **A** “Human PBMC lupus”: 8 lupus patients from batch 1 under the same condition. **B** “Mouse brain FC” major cell types: 7 mouse subjects from the same frontal cortex region under the same condition. **C** “Mouse brain FC” sub-cell types: 7 mouse subjects from the same frontal cortex region under the same condition. Down-sample boxes in **A**–**C** each contains 30 results. **D** “Mouse brain FC” to predict “Mouse brain pFC” on major cell types: use 7 mouse subjects respectively from “Mouse brain FC” as a reference to predict 6 mouse subjects from “Mouse brain pFC”. Individual effect box contains 42 results; down-sample box contains 60 results, and “pooling” box contains 6 results

As shown in Fig. 5A–C, under intra-dataset setting, combining individuals together (black line) achieves significantly higher overall accuracy compared to the other two strategies in all datasets. The same trends can be observed in ARI and macroF1 (Additional file 1: Figure S7A-C). As for the down-sampling strategy, we can also observe a slight increase in the mean performance with lower variance, indicating that the benefit of pooling is not only from the increased reference data size. Another finding from the figures is the significant increase in performance when predicting sub-cell types in the mouse brain dataset (Fig. 5C). This indicates that, for a large number of sub-cell types, an increased sample size is particularly beneficial. Figure 5D and Additional file 1: Figure S7D show the comparison under the inter-dataset setting, where “pooling” brings slightly better performances, similar to that in the intra-dataset experiments.

#### Pooling reference from different conditions can improve the prediction results

Next, we wonder how “pooling” subjects with different conditions will impact the prediction performance. We combine subjects from different brain regions and different datasets in mouse brain data, as well as individuals from different batches and clinical conditions in human PBMC. For the dataset effect, we first try to combine individuals in each dataset respectively and then merge the two datasets together to predict.

All accuracy and Macro F1 metrics are improved by combining individuals together (Table 1), although some experiments have slight drops in ARI. These results indicate that by “pooling” individuals together, some noises caused by individual variations can be averaged out. We also find when combining “Mouse brain pFC” and “Mouse brain cortex” to predict the target in “Mouse brain FC,” we can achieve a better result in Macro F1 than combining individuals from “Mouse brain pFC” and “Mouse brain cortex,” respectively. We further visualize the cell type annotations after combining the “Mouse brain pFC” and “Mouse brain cortex” using tSNE [48]. We observe the two cortex datasets actually do not blend well; instead, a cluster of interneurons and neurons from “Mouse brain cortex” is mixed together (Additional file 1: Figure S8). Even though there is a clear separation between datasets as shown in these tSNE plots, our results show that combining datasets can still improve the prediction performance.

**Table 1:** Before and after “pooling”

	Before (mean Acc)	After (Acc)	Before (mean ARI)	After (ARI)	Before (mean Macro F1)	After (Macro F1)
Mouse brain region effect	0.993	**0.996**	0.995	**0.997**	0.915	**0.959**
Mouse brain dataset effect (pFC)	0.971	**0.988**	0.947	**0.967**	0.892	**0.904**
Mouse brain dataset effect (cortex)	0.977	**0.982**	0.955	**0.965**	0.918	**0.927**
Mouse brain dataset effect (combine pFC and cortex)	–	**0.986**	–	**0.963**	–	**0.933**
Human PBMC lupus batch effect	0.872	**0.893**	0.779	**0.789**	0.717	**0.790**
Human PBMC lupus clinical difference	0.838	**0.850**	0.724	0.716	0.673	**0.701**
Human PBMC lupus (combine batch effect and clinical difference)	–	**0.896**	–	**0.782**	–	**0.790**

Performance comparisons between before and after “pooling” individuals with condition effect. “–” indicates the data is unavailable. The bold data indicates a performance improvement

After showing that pooling reference data can improve prediction performance, we wonder if there is a saturation point when we keep enlarging the reference datasets. We conduct three analyses in mouse brain data to investigate the pooling saturation point on three perspectives: (1) predict major cell type within the same dataset, (2) predict major cell type across different datasets, and (3) predict sub-cell type within the same dataset. The saturation analyses are done in two ways. We first combine cells from different numbers of individuals as a reference. Furthermore, we pool the cells from all individuals and randomly sample different numbers of cells as a reference. The second averages out the individual effects and only investigates whether there will be saturation with more cells in the reference. More details about datasets used in this analysis are provided in Additional file 1: Section S2, and analyses details are provided in Additional file 1: Section S3.

We notice that for major cell type prediction (Additional file 1: Figure S9A, B), performance saturation clearly exists with larger reference data. For sub-cell type prediction (Additional file 1: Figure S9C), we do not observe a clear saturation point, and it is likely that the performance can further improve with larger reference. The low signal-to-noise ratio among the subtypes requires an even larger reference for us to observe the saturation. Another finding from this analysis is that pooling individuals can potentially lead to faster saturation. This is very pronounced in sub-cell type prediction (Additional file 1: Figure S9B). The right panel shows that the performance is saturated from the start (3000 cells) when cells are sampled from a pool of individuals. When adding each individual at a time (left panel), it requires 4 individuals (around 40,000 cells) to reach saturation. These results are consistent with our findings that pooling individuals can achieve better prediction performance.

### Purifying references does not improve the prediction results

Furthermore, we investigate whether purifying the reference dataset can achieve better predictions. Intuitively, cells on the edge of the cluster can be easily misclassified, and including them in the reference can contaminate the signals. We adopt two strategies for purifying the reference data: (1) Euclidean distance-based and (2) probability-based. The distance-based purification first computes the centroids for each cell cluster and then removes the 10% of cells with the largest distance to the centroid. For probability-based purification, we first adopt an SVM classifier with RBF kernel to fit the reference data and then generate probability scores of each cell belonging to cell types. For each cell type, 10% of cells with the lowest probability scores are removed. We conduct both purifications in four designed analyses. More details can be found in Additional file 1: Section S2.

We first visualize those cells removed from the reference dataset (Additional file 1: Figure S10) and find that distance-based purification evenly removes cells on the edge of the clusters while probability-based purification removes more cells lying in between different clusters. Table 2 presents the overall accuracies of the four comparisons before and after purifications. The results vary in different analyses. In predicting mouse brain sub-cell types, both purifications only lead to slightly improved performances. The reason might be that the purification removes wrongly labeled cells and increases the separations among cell clusters. Overall, cell purification does not improve the performance when predicting major cell types because the outliers of cell clusters do not have a large impact on assigning labels. However, when there exist sub-cell types, outliers among cell clusters act as noises, and by removing those, the prediction can be slightly improved.

**Table 2:** Before and after “purification”

	Original performance	After distance-based purification	After probability-based purification
Human PBMC lupus: one individual predicts another individual	0.797	0.748	**0.799**
Human PBMC lupus: one batch predicts another batch	0.924	0.920	0.921
Human PBMC lupus: one status predicts another status	0.931	**0.932**	**0.934**
Mouse brain FC: one subject predicts another subject (sub-cell types)	0.783	**0.813**	**0.802**

Performance comparisons before and after purifications on reference dataset. Here, we only demonstrate the performance of overall accuracy. There are in total four experiments: (1) “Human PBMC lupus” one individual predicts another individual: uses one lupus patient from batch 1 to predict another from the same batch under the same condition; (2) “Human PBMC lupus” one batch predicts another batch: uses samples from batch 1 to predict samples from batch 2 under the same condition; (3) “Human PBMC lupus” one status predicts another status: uses lupus samples from batch 2 to predict IFN-β stimulated samples from the same batch; and (4) “Mouse brain FC” one subject predicts another subject: uses one mouse subject to predict sub-cell types of another subject from the same region and the same dataset. The bold data indicates a performance improvement

### Predicting sub-cell types

For most of the above experiments, we focus on predicting major cell types. Predicting sub-cell types is much more complicated due to several difficulties. First of all, the definitions of subtypes across datasets are not consistent. Take the mouse brain dataset as an example, the subtypes are identified by two-stage independent component analysis (ICA) in the original paper [36]. Among the subtypes, there are 29 neuron clusters labeled with layer information (i.e., layers 2–3, layer 5, layer 6) in the frontal cortex region while 44 neuron clusters without layer information in the hippocampus. These neuron subtypes are not comparable across the brain regions nor across datasets. Even when the subtype definitions are consistent, e.g., predicting within the same brain region in the same dataset, the prediction accuracy for subtypes is much lower (Fig. 5C). If one would like to predict subtypes using a supervised method, there has to be comprehensive and consistent subtype definitions for different tissue types and preferably under different conditions. Along with the rapid accumulation of single-cell experiments, for example, from large consortia such as Human Cell Atlas [49], we believe such data will be available in the near future. When subtype definition is not available, we recommend a two-step approach: first, predict the major cell types using a supervised method, then an unsupervised clustering method for subtypes under each major cell type separately. Such a strategy also requires a number of other methodological considerations, including the feature selection strategy in each major cell type and new/rare subtype identification. Those are our research plans for the near future.

### Predicting novel cell types

In general, the supervised methods are not designed for discovering unseen labels. Without the additional procedure, any new cell type in the target data will be forced into one of the existing cell types in the reference. However, it is possible to set a threshold on the prediction probabilities to classify some cells as *unassigned*, which is the strategy used in scmap and CHETAH. The new cell types can hopefully be classified as *unassigned* for further analyses to identify new cell types. We perform additional analyses to investigate such a strategy on MLP. Briefly speaking, we first remove one or two cell types from the reference dataset (thus, there are “new” cell types in the target data) and then do prediction. We wish that cells in the “new” cell type(s) have a lower prediction probability for not belonging to any existing cell types, thus will be classified as *unassigned*. The results from four datasets (Additional file 1: Figure S11) show that such an approach can reasonably call new cell types as *unassigned*. Details about the procedure and results are presented in Additional file 1: Section S4. Of course, calling *unassigned* cell types is not equal to identifying new cell types, but it is the first step. How to use supervised methods to identify new cell types is an interesting and important question and is our research topic in the near future.

### Computational performance

Besides prediction performances, we also keep records of the training time for each experiment.

**Fig. 6:**
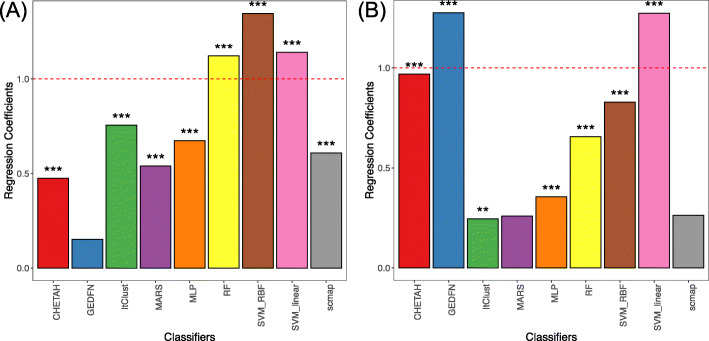
Computation performance of each method. The horizontal dotted red line denotes 1 and indicates a linear relation. The star denotes the *p*-value of the estimates (**p-value < 0.01; ***p-value < 0.001). **A** Regression coefficients of each method describe the relationship between training time and reference data size. As shown in the figure, the training time of SVM and random forest grows faster than the increase of reference data size, and all others are slower. Among all classifiers, the coefficient estimation of GEDFN is not significant. **B** Regression coefficients of each method describe the relationship between training time and number of cell types. The training time of GEDFN and SVM with linear kernel grows faster than the increase of the number of cell types. Coefficient estimations of scmap and MARS are not significant

The training time can be affected by both reference size and number of cell types, which are moderately positively correlated with the Pearson correlation coefficient being 0.44. To fully evaluate how training time is affected by each classifier, we construct a linear regression model using the log-transformed training time *t* as a response and the log-transformed reference size *s* along with the log-transformed number of cell types *c* as explanatory variables. This regression model can be further denoted as log(*t*)~*β*_1_ log(*s*) + *β*_2_ log(*c*). We estimate *β* for each classifier. If *β* is greater than 1, the training time grows faster than linear and vice versa. Regression coefficients are summarized in Fig. 6. It shows that RF and SVM with both kernels have the worst computational performances in terms of training data size, and GEDFN and SVM (linear kernel) are the worst in terms of the number of cell types. Overall, ItClust and MLP show the best scalability with the coefficients of both reference size and number of cell types are less than 1. This might be caused by the design of the loss function which directly takes all classes into account. Compared to scmap, another correlation-based method, CHETAH, is largely affected by the number of cell types because it adopts a hierarchical structure and needs to derive gene profiles for each branch until discovering all cell types. With these observations, we again promote the usage of MLP with its comparably better performance and high scalability. When “pooling” all cells together, training an MLP classifier will not consume too much training time. Once the classifier is trained, parameters can be stored and directly used for predicting cells in the newly generated scRNA-seq datasets. Prediction can be done in a very short time period.

## Discussion

Supervised cell typing for scRNA-seq has gained tremendous interest in recent years, and we believe it is the direction to go for identifying cell types in scRNA-seq data. In this paper, we comprehensively evaluate several important aspects of supervised cell typing: feature selection, prediction method, data preprocessing, and the impact of discrepancies between reference and target dataset, which are important choices to make for investigators. Even though there are a number of methods and several comparison studies, no one has investigated the combined effects of these procedures, in particular, the choice of reference datasets. Moreover, we also investigate the strategies of processing reference data, including reference pooling and purification.

Based on our results, we make the following main recommendations. First, applying *F*-test on the reference dataset to select features and using MLP as a classifier is the best performer overall when the reference data is reasonably large (e.g., > 5000 cells). In fact, MLP can be replaced by SVM with either linear or RBF kernel, which produces comparable results. However, due to the computational burden of training SVM, especially in large datasets or having many cell types, we recommend using MLP. When the reference data is small, using a correlation-based method such as scmap is a better strategy. We consider certain pre-processing (e.g., imputation and batch effect correction) steps unnecessary because we do not observe a significant performance increase by doing so. Secondly, it is always desirable to pick reference data with matching biological and clinical conditions with the target data. However, the discrepancy between the reference and target data only has a slight impact on predicting major cell types. Thus, it is not terribly bad to use a reference dataset with slight condition differences, even though significant clinical differences could lead to non-trivial performance reductions. Thirdly, pooling reference from different datasets improves the prediction results. This is not only because of the increased reference data size, but also because that pooling can average out some biological and technical variations (evidenced by our downsampling prediction results and pooling saturation analysis). In the pooling saturation analyses, we sometimes observe that adding certain cells or individuals may result in a worse performance (Additional file 1: Figure S9C). This leads to an important question on how to assess and select high-quality references. This is beyond the scope of this work, but we will explore it in the near future. Moreover, we find that purifying the reference data does not significantly improve the results, so we recommend against such a procedure.

From our investigations, the major cell type prediction is a relatively easier task as all analyses achieve satisfying results. However, supervised prediction for sub-cell types is much more difficult mainly due to the inconsistent subtype definition from different datasets. In fact, it is highly possible that the sub-cell types are indeed different under distinct biological and clinical conditions. Due to these reasons, we recommend against supervised prediction for sub-cell types and suggest a two-step hybrid approach: first applying a supervised prediction for major cell types and then using unsupervised approaches for subtypes. This might require further development and evaluation of the unsupervised clustering methods for similar sub-cell types, since most current methods focus on clustering major cell types. When the goal of an unsupervised method is to distinguish many very similar cell subtypes, we might need new algorithms for feature selection, cell clustering, and new/rare subtype identification. It is worth mentioning that even though the two semi-supervised methods we test (ItClust and MARS) do not perform as well as the supervised ones in predicting known cell types, they have the potential advantage to discover new cell types, which could be useful in subtype prediction.

## Conclusions

With the increased application of scRNA-seq, especially in large-scale, population-level studies, cell type identification continues to be one of the most important questions in scRNA-seq data analysis, for which we believe the supervised cell typing method will be a better answer. We perform extensive evaluations on several important factors in such an approach and provide some recommendations. While existing benchmark studies directly compare available “off-the-shelf” supervised tools, we take a step further to evaluate combinations of different strategies. More importantly, we evaluate the impact of the reference data and potential strategies for processing reference data, which have never been done before. Our study not only provides performance evaluation and recommendation, but also points out potential research directions in this field.

## Methods

### Implementation of feature selection

For “no feature selection” strategy, we directly find the common genes between reference and target dataset. For selecting features by Seurat, we use the function scanpy.pp.highly_variable_genes() from SCANPY [50] Python package (version: 1.5.0) with flavor as “seurat” to select features on both reference and target. For FEAST and *F*-test, we use the FEAST R package (version: 0.1.0, https://github.com/suke18/FEAST). When applying FEAST, we first use the Consensus() function to get the consensus clustering results and then use cal_F2() to calculate the *F* statistics on both reference and target. As for the *F*-test, we directly use the cal_F2() function with annotated cell clusters given by the reference dataset.

### Implementation of cell typing classifiers

In total, we have 9 classifiers. scmap (version 1.8.0) and CHETAH (version 1.2.0) have available R packages, and others are implemented in Python. We skip the original built-in feature selection procedure and provide our selected features.

For scmap, we use the scmapCluster() function from the scmap package to perform the analysis. To remove “unassigned” cells, we set the threshold as 0 to force full assignment. For CHETAH, we first perform a dimension reduction on our selected features as required, and then use CHETAHclassifier() to build the classifier. Then, we set the threshold as 0 in Classify() to force full assignment.

To build the machine learning models, we use the scikit-learn (version: 0.23.2) package. We use sklearn.svm.SVC() with “rbf” kernel for SVM RBF, sklearn.svm.LinearSVC() for SVM linear, and sklearn.ensemble.RandomForestClassifier() with 50 estimators for random forest. For SVM classifiers, we use “one-versus-rest” scheme.

To realize deep learning approaches, we use TensorFlow (version 1.15.0) [51] as a core for MLP, GEDFN, and ItClust, and use PyTorch (version 1.2.0) [52] as a core for MARS. For MLP, we use Dense layers with rectified linear unit (ReLU) activation function, and we apply dropout after each layer with a dropout rate of 0.1. Cross-entropy loss is used for training the model. For GEDFN (https://github.com/yunchuankong/GEDFN), we modify its original code with our designed input and output. We embed a protein-protein interaction network from the HiNT database [53]. For ItClust (https://github.com/jianhuupenn/ItClust), we substitute Louvain clustering with *k*-means and set *K* as the number of cell types in the reference dataset in order to achieve the desired number of clusters.

The network structures of MLP, GEDFN, and ItClust are dependent on the sample size. When there are over 5000 cells, MLP has the structure of [input_dim, 128, 64, 32, 16, 8, n_classes] (input_dim refers to the length of feature space, n_classes refers to the number of cell types), GEDFN has [input_dim, input_dim, 128, 32, n_classes], and ItClust has [input_dim, 128, 32] where 32 is the latent space dimension of the autoencoder model. When there are less than 5000 cells, MLP has [input_dim, 64, 16, n_classes], GEDFN has [input_dim, input_dim, 16, n_classes], and ItClust has [input_dir, 16] where 16 is the latent dimension. For MARS, we adopt the default settings with the structure of [input_dim, 1000, 100] in the autoencoder model and use the default train-validation split (90% as training and 10% as validation) to fine-tune the model. We train deep learning models using Titan RTX on all experiments, but in order to test the scalability of MLP, we also run several analyses with different reference sizes and numbers of cell types using MLP on CPU to compare the runtime. It turns out the runtime of MLP for using GPU and CPU are comparable, and when the data is small, training on CPU is even faster. However, for more complex networks such as GEDFN, ItClust, and MARS, training on GPU can be three times faster.

### Implementation of imputation

MAGIC (https://github.com/KrishnaswamyLab/MAGIC) is both available in Python and R. Here, we use Python implementation (version 3.0.0) to accommodate our Python pipeline. After creating the object by magic.MAGIC() function, we use fit_transform() function to impute the data. scVI (https://github.com/YosefLab/scvi-tools, version 0.11.0) is also available in Python, and we use PyTorch (version 1.8.0) as a core. We use the scvi.data.setup_anndata() function to initiate data and then use the scvi.model.SCVI() function to create an scVI model.train() function is then called to train the model and get_normalized_expression() function is used to acquire imputed data. SAVER (version 1.1.2) is available in R and saver() function with the number of cores as 10 is applied to impute the data.

### Implementation of batch effect correction

Harmony (version 1.0) and fastMNN (version 1.2.4) are both implemented in R. We consider reference as one batch and target as another, then correct for the discrepancies. In Harmony, we first reduce the data dimension by performing a principal component analysis (PCA) with the irlba (https://cran.r-project.org/web/packages/irlba/index.html, version 2.3.3) R package to top 20 PCs. Then, the HarmonyMatrix() function is called to remove the batch effect between reference and target. Because Harmony does not offer a corrected gene expression matrix, we directly use the corrected values from PCA reduction space as reference and target for cell type prediction. In fastMNN, we directly use the fastMNN() function to correct for batch effect and extract out the corrected values for prediction.

## Supplementary Information

**Additional file 1.** Supplementary note section S1-S5, Table S1-S3 and Figures S1-S16.

**Additional file 2. **Overview of experiments Excel *Results_Summary_Table.xlsx* (Excel file).

**Additional file 3. **R data frame including all 29 experiment results *experiment_results.RDS* (an R RDS file).

**Additional file 4.** Review history.

## Data Availability

The datasets analyzed during the current study are available in the Gene Expression Omnibus (GEO), Single Cell Portal (SCP), ArrayExpress, NeMO, and 10X Genomics platform with the following accession numbers: mouse brain FC/HC [36] (GSE116470), mouse brain pFC [37] (GSE124952), mouse brain cortex [32] (SCP425), mouse brain Allen [38] (NeMO: dat-jb2f34y), human PBMC lupus [31] (GSE96583), human PBMC protocols [32] (SCP424), human PBMC FACS [39] (10X Genomics Datasets under “Single Cell 3′ Paper: Zheng et al. 2017”), human Pancreas Muraro [33] (GSE85241), human pancreas Segerstolpe [34] (E-MTAB-5061), and human pancreas Xin [35] (GSE81608). The analysis code is freely available on GitHub (https://github.com/marvinquiet/RefConstruction_supervisedCelltyping) [54] and Zenodo (10.5281/zenodo.5237218) [55]. The source code is released under MIT license.

## References

[1] Hwang B, Lee JH, Bang D (2018). Single-cell RNA sequencing technologies and bioinformatics pipelines. Exp Mol Med.

[2] Haque A, Engel J, Teichmann SA, Lönnberg T (2017). A practical guide to single-cell RNA-sequencing for biomedical research and clinical applications. Genome Med.

[3] Nadal-Ribelles M, Islam S, Wei W, Latorre P, Nguyen M, de Nadal E, Posas F, Steinmetz LM (2019). Sensitive high-throughput single-cell RNA-seq reveals within-clonal transcript correlations in yeast populations. Nat Microbiol.

[4] Li B, Severson E, Pignon J-C, Zhao H, Li T, Novak J (2016). Comprehensive analyses of tumor immunity: implications for cancer immunotherapy. Genome Biol.

[5] Merienne N, Meunier C, Schneider A, Seguin J, Nair SS, Rocher AB (2019). Cell-type-specific gene expression profiling in adult mouse brain reveals normal and disease-state signatures. Cell Rep.

[6] Jindal A, Gupta P, Sengupta D (2018). Discovery of rare cells from voluminous single cell expression data. Nat Commun.

[7] Trapnell C (2015). Defining cell types and states with single-cell genomics. Genome Res.

[8] Davey HM, Kell DB (1996). Flow cytometry and cell sorting of heterogeneous microbial populations: the importance of single-cell analyses. Microbiol Rev.

[9] Kiselev VY, Kirschner K, Schaub MT, Andrews T, Yiu A, Chandra T, Natarajan KN, Reik W, Barahona M, Green AR, Hemberg M (2017). SC3: consensus clustering of single-cell RNA-seq data. Nat Methods.

[10] Butler A, Hoffman P, Smibert P, Papalexi E, Satija R (2018). Integrating single-cell transcriptomic data across different conditions, technologies, and species. Nat Biotechnol.

[11] Kiselev VY, Yiu A, Hemberg M (2018). scmap: projection of single-cell RNA-seq data across data sets. Nat Methods.

[12] Alquicira-Hernandez J, Sathe A, Ji HP, Nguyen Q, Powell JE (2019). scPred: accurate supervised method for cell-type classification from single-cell RNA-seq data. Genome Biol.

[13] de Kanter JK, Lijnzaad P, Candelli T, Margaritis T, Holstege FC (2019). CHETAH: a selective, hierarchical cell type identification method for single-cell RNA sequencing. Nucleic Acids Rese.

[14] Pliner HA, Shendure J, Trapnell C (2019). Supervised classification enables rapid annotation of cell atlases. Nat Methods.

[15] Chen L, He Q, Zhai Y, Deng M. Single-cell RNA-seq data semi-supervised clustering and annotation via structural regularized domain adaptation. Bioinformatics. 2021;37(6):775–84. 10.1093/bioinformatics/btaa908. 10.1093/bioinformatics/btaa908 33098418

[16] Hu J, Li X, Hu G, Lyu Y, Susztak K, Li M (2020). Iterative transfer learning with neural network for clustering and cell type classification in single-cell RNA-seq analysis. Nat Mach Intell.

[17] Brbić M, Zitnik M, Wang S, Pisco AO, Altman RB, Darmanis S, Leskovec J (2020). MARS: discovering novel cell types across heterogeneous single-cell experiments. Nat Methods.

[18] Lin W-C, Tsai C-F, Hu Y-H, Jhang J-S (2017). Clustering-based undersampling in class-imbalanced data. Inf Sci.

[19] Kiselev VY, Andrews TS, Hemberg M (2019). Challenges in unsupervised clustering of single-cell RNA-seq data. Nat Rev Genet.

[20] Abdelaal T, Michielsen L, Cats D, Hoogduin D, Mei H, Reinders MJ (2019). A comparison of automatic cell identification methods for single-cell RNA sequencing data. Genome Biol.

[21] Huang Q, Liu Y, Du Y, Garmire LX. Evaluation of cell type annotation R packages on single-cell RNA-seq data. Genomics Proteomics Bioinform. 2020. 10.1016/j.gpb.2020.07.004. 10.1016/j.gpb.2020.07.004 PMC8602772 33359678

[22] Stuart T, Butler A, Hoffman P, Hafemeister C, Papalexi E, Mauck WM (2019). Comprehensive integration of single-cell data. Cell.

[23] Pasquini G, Arias JER, Schäfer P, Busskamp V. Automated methods for cell type annotation on scRNA-seq data. Comput Struct Biotechnol J. 2021;19:961–9. 10.1016/j.csbj.2021.01.015. PMC7873570 33613863

[24] Pedregosa F, Varoquaux G, Gramfort A, Michel V, Thirion B, Grisel O (2011). Scikit-learn: machine learning in Python. J Mach Learn Res.

[25] Rumelhart DE, Hinton GE, Williams RJ (1986). Learning representations by back-propagating errors. Nature.

[26] Kong Y, Yu T (2018). A graph-embedded deep feedforward network for disease outcome classification and feature selection using gene expression data. Bioinformatics.

[27] Guo H, Li J (2021). scSorter: assigning cells to known cell types according to marker genes. Genome Biol.

[28] Tan Y, Cahan P (2019). SingleCellNet: a computational tool to classify single cell RNA-Seq data across platforms and across species. Cell Syst.

[29] Zhang AW, O’Flanagan C, Chavez EA, Lim JL, Ceglia N, McPherson A (2019). Probabilistic cell-type assignment of single-cell RNA-seq for tumor microenvironment profiling. Nat Methods.

[30] Su K, Yu T, Wu H. Accurate feature selection improves single-cell RNA-seq cell clustering. Brief Bioinform. 2021. 10.1093/bib/bbab034. 10.1093/bib/bbab034 PMC8644062 33611426

[31] Kang HM, Subramaniam M, Targ S, Nguyen M, Maliskova L, McCarthy E (2018). Multiplexed droplet single-cell RNA-sequencing using natural genetic variation. Nat Biotechnol.

[32] Ding J, Adiconis X, Simmons SK, Kowalczyk MS, Hession CC, Marjanovic ND, Hughes TK, Wadsworth MH, Burks T, Nguyen LT, Kwon JYH, Barak B, Ge W, Kedaigle AJ, Carroll S, Li S, Hacohen N, Rozenblatt-Rosen O, Shalek AK, Villani AC, Regev A, Levin JZ (2020). Systematic comparison of single-cell and single-nucleus RNA-sequencing methods. Nat Biotechnol.

[33] Muraro MJ, Dharmadhikari G, Grün D, Groen N, Dielen T, Jansen E (2016). A single-cell transcriptome atlas of the human pancreas. Cell Syst.

[34] Segerstolpe Å, Palasantza A, Eliasson P, Andersson E-M, Andréasson A-C, Sun X, Picelli S, Sabirsh A, Clausen M, Bjursell MK, Smith DM, Kasper M, Ämmälä C, Sandberg R (2016). Single-cell transcriptome profiling of human pancreatic islets in health and type 2 diabetes. Cell Metab.

[35] Xin Y, Kim J, Okamoto H, Ni M, Wei Y, Adler C, Murphy AJ, Yancopoulos GD, Lin C, Gromada J (2016). RNA sequencing of single human islet cells reveals type 2 diabetes genes. Cell Metab.

[36] Saunders A, Macosko EZ, Wysoker A, Goldman M, Krienen FM, de Rivera H (2018). Molecular diversity and specializations among the cells of the adult mouse brain. Cell.

[37] Bhattacherjee A, Djekidel MN, Chen R, Chen W, Tuesta LM, Zhang Y (2019). Cell type-specific transcriptional programs in mouse prefrontal cortex during adolescence and addiction. Nat Commun.

[38] Yao Z, van Velthoven CTJ, Nguyen TN, Goldy J, Sedeno-Cortes AE, Baftizadeh F (2021). A taxonomy of transcriptomic cell types across the isocortex and hippocampal formation. Cell.

[39] Zheng GX, Terry JM, Belgrader P, Ryvkin P, Bent ZW, Wilson R (2017). Massively parallel digital transcriptional profiling of single cells. Nat Commun.

[40] Hou W, Ji Z, Ji H, Hicks SC (2020). A systematic evaluation of single-cell RNA-sequencing imputation methods. Genome Biol..

[41] Van Dijk D, Sharma R, Nainys J, Yim K, Kathail P, Carr AJ (2018). Recovering gene interactions from single-cell data using data diffusion. Cell.

[42] Huang M, Wang J, Torre E, Dueck H, Shaffer S, Bonasio R, Murray JI, Raj A, Li M, Zhang NR (2018). SAVER: gene expression recovery for single-cell RNA sequencing. Nat Methods.

[43] Lopez R, Regier J, Cole MB, Jordan MI, Yosef N (2018). Deep generative modeling for single-cell transcriptomics. Nat Methods.

[44] Tran HTN, Ang KS, Chevrier M, Zhang X, Lee NYS, Goh M (2020). A benchmark of batch-effect correction methods for single-cell RNA sequencing data. Genome Biol.

[45] Korsunsky I, Millard N, Fan J, Slowikowski K, Zhang F, Wei K, Baglaenko Y, Brenner M, Loh PR, Raychaudhuri S (2019). Fast, sensitive and accurate integration of single-cell data with Harmony. Nat Methods.

[46] Haghverdi L, Lun AT, Morgan MD, Marioni JC (2018). Batch effects in single-cell RNA-sequencing data are corrected by matching mutual nearest neighbors. Nat Biotechnol.

[47] Lun A (2019). Further MNN algorithm development.

[48] van der Maaten L, Hinton G (2008). Visualizing data using t-SNE. J Mach Learn Res.

[49] Regev A, Teichmann SA, Lander ES, Amit I, Benoist C, Birney E, et al. The Human Cell Atlas. Elife. 2017;6. 10.7554/eLife.27041. 10.7554/eLife.27041 PMC5762154 29206104

[50] Wolf FA, Angerer P, Theis FJ (2018). SCANPY: large-scale single-cell gene expression data analysis. Genome Biol.

[51] Abadi M, Agarwal A, Barham P, Brevdo E, Chen Z, Citro C, et al. Tensorflow: large-scale machine learning on heterogeneous distributed systems. arXiv preprint arXiv. 2016;arXiv:1603.04467.

[52] Paszke A, Gross S, Massa F, Lerer A, Bradbury J, Chanan G, et al. Pytorch: an imperative style, high-performance deep learning library. arXiv preprint arXiv. 2019;arXiv:1912.01703.

[53] Patil A, Nakamura H (2005). HINT: a database of annotated protein-protein interactions and their homologs. Biophysics.

[54] Wenjing M, Kenong S, Hao W. Reference construction strategies for single-cell supervised cell typing source code. GitHub. 2021. https://github.com/marvinquiet/RefConstruction_supervisedCelltyping.

[55] Wenjing M, Kenong S, Hao W. Reference construction strategies for single-cell supervised celltyping source code. Zenodo. 2021. 10.5281/zenodo.5237218.

