# Supplementary material for: Evaluation of some aspects in supervised cell type identification for single-cell RNA-seq: classifier, feature selection, and reference construction
Source: Genome Biol. 2021 Sep 9;22:264. doi: 10.1186/s13059-021-02480-2 (PMC8427961; doi:10.1186/s13059-021-02480-2)
Supplement: Supplementary file 1 — Additional file 1. Supplementary note section S1-S5, Table S1-S3 and Figures S1-S16. [file 13059_2021_2480_MOESM1_ESM.docx]

**Additional file 1**

**S1. Performance Grain / Loss Calculation**

For each metric in each experiment, we have 9 classifiers and 6 feature selection methods resulting in a 9x6 two-way table. We first calculate the mean of each two-way table as the experiment’s baseline performance. Next, we subtract the mean from the two-way table for each experiment and aggregate all experiments together by taking the average. This results in one 9x6 data matrix for each metric. Finally, we sort the table in a descending way by rows and columns, in which the top left corner combination has the most gain while bottom right combination has the most loss. We present the gains/losses in a heatmap for each metric.

When merely focusing on the gains/losses from feature selection strategies, we subtract the mean of each column (representing each feature selection method) to average out effects from classifiers. Then, we combine all results together to draw the boxplot. Similar procedure has been done for evaluating the gains/losses from different classifiers.

**S2. Data Pre-processing**

All scRNA-seq datasets have been pre-processed by filtering out low-quality cells expressing in less than 10 genes and genes expressing in less than 10 cells. We then use normalize each cell to have 10,000 reads and do log-transformation. Next, we scale the dataset to zero mean and unit variance and truncate absolute values with maximum of 6. Finally, the data is fed to corresponding classifiers.

***Datasets for comparing cell type annotations***

To assess the effects of choosing between computationally derived annotations and fluorescence-activated cell sorting (FACS) datasets as target, we conduct four experiments using “Human PBMC FACS” as target. Because FACS data is usually generated based on experts’ *a priori* knowledge of tissue composition, cell types captured by FACS are not comprehensive. For example, in the “Human PBMC FACS” dataset, CD16+ Monocytes, Megakaryocytes and Dendritic cells are not included. We first perform two cross-validation predictions (80% as reference and the rest 20% as target). One uses original 10 cell subpopulations captured by FACS data and the other uses curated cell types which are common in other datasets. Our curation process merges CD4+ T sub-cell types into CD4+T cells, merges CD8+ T sub-cell types into CD8+ T cells and removes CD34+ cells which results into 5 major cell populations. In addition, we perform two cross dataset predictions. One uses a lupus patient (ID: 1154) from “Human PBMC lupus” batch1 as reference and the other uses pbmc2 fresh sample profiled by 10X Chromium from “Human PBMC protocols” as reference. The results of the above four experiments are aggregated by calculating the performance gain/loss (Figure S3).

***Datasets for comparing condition effects***

For comparing condition effects, we include 7 mice from “Mouse brain FC” and 6 mice from “Mouse brain HC” using Drop-seq, 6 mice with saline treatment from “Mouse brain pFC” using 10X Chromium, and 2 cortex samples named cortex1 and cortex2 from “Mouse brain cortex” using DroNc-seq. DroNc-seq and Drop-seq are proved to have similar performance [1]. To summarize the differences for the two datasets effect, the “Mouse brain pFC” contains protocol difference and the “Mouse brain cortex” includes certain region difference as it profiles the whole cortex region.

We then curate each dataset to contain only major cell types, such as integrating multi-layers of neurons in “Mouse brain FC” together as neurons, removing pericytes from “Mouse brain cortex”, etc. During cell type curation, we find there exists both newly formed oligodendrocytes (NF Oligo) and oligodendrocytes (Oligo) in “Mouse brain pFC”. We first categorize NF Oligo into oligodendrocytes, but we find NF Oligo is annotated as polydendrocytes in “Mouse brain FC” when visualizing subjects from “Mouse brain FC” and “Mouse brain pFC” together (Figure S14). Therefore, we decide to categorize NF Oligo as polydendrocytes. In summary, there are 7 major cell types: neuron, interneuron, astrocytes, oligodendrocyte, polydendrocyte, endothelial and microglia. We use P60FCCx3cr1Rep1 from “Mouse brain FC” as the target individual to validate the condition effect.

***Datasets for comparing pooling effect***

Under intra-dataset setting, we conduct three experiments (1) 8 lupus patients in batch1 from “Human PBMC lupus”, (2) 7 mice from “Mouse brain FC” using 14 major cell types, and (3) the same subjects using 81 sub-cell types. We fix one individual (ID: 1085) in (1) and one mouse subject (P60FCCx3cr1Rep1) in (2)(3) as target and then perform the “pooling” strategy. For “pooling”, we combine all other individuals or subjects together to predict the fixed one. Then, we down-sample the combined reference to the average number of the dataset (total number of cells divided by number of individuals or subjects) for 30 times. As for inter-dataset, we use 7 mice in “Mouse brain FC” to predict 6 mice with saline treatment in “Mouse brain pFC”. Curation procedure has been done first. Then, we use each mouse in “Mouse brain pFC” as target to perform individual effect and then the “pooling” strategy. When using each mouse as target, the down-sampling is performed 10 times.

***Datasets for pooling saturation analysis***

When analyzing the performance saturation of using larger reference data, we conduct three experiments using mouse brain datasets because they have more individuals and cells compared to other datasets. For predicting major and sub- cell types within the dataset, we use 6 individuals in “Mouse brain FC” to predict the rest individual (P60FCCx3cr1Rep1). For across datasets prediction, we use all 6 mice from “Mouse brain pFC” and 3 mice from “Mouse brain Allen” to predict major cell types in one mouse from “Mouse brain FC” (P60FCCx3cr1Rep1) to mimic the real scenario.

***Datasets for purifications***

We conduct four experiments for testing cell purifications. First three experiments come from “Human PBMC lupus” and the last experiment is conducted on “Mouse brain FC”. We (1) use one lupus patient (ID: 1154) to predict another patient (ID: 1085) in batch1; (2) use 8 lupus samples from batch1 to predict 8 lupus samples from batch2; (3) use 8 lupus samples from batch2 to predict 8 IFN-β treated samples from the same batch; (4) use one mouse subject (ID: P60FCRep1) to predict another subject (ID: P60FCCx3cr1Rep1) from the same brain region on sub-cell types. For distance-based purification, we first compute each cluster’s centroid by averaging the processed read count matrix (scale and log-normalized) of cells belonging to this cluster. Next, we compute the Euclidean distance between each cell and the centroid, and remove 10% cells with largest distance. For probability-based purification, we first fit an SVM with RBF kernel model on the reference dataset and generate a probability matrix denoting how possible a cell belongs to a cluster. Then, for each cluster, we remove 10% cells with the lowest probability. After purifying the cluster, we predict again on the same target dataset.

**S3. Analyses details on pooling saturation**

For creating reference data by combining individuals, we first randomly shuffle the orders of the individuals, and then sequentially add them to reference dataset. For each reference, we perform F-test to select top 1000 features selection and predict cell types in the target dataset. To remove the potential variations brought by the order of the individual, we repeat the above procedure for 50 times and average the results. For creating reference data by subsampling from all cells, we first pool all cells from all individuals together and randomly shuffle the order. We add 3,000 cells each time to create reference dataset. Again, we repeat this process for 50 times in order to reduce the variations in sampling.

**S4. Analyses details on new cell type discovery**

Due to the intrinsically different models underlying each classifier, it is unreasonable to select a uniform threshold to benchmark all. Therefore, we apply the thresholding scheme in MLP classifier as an example to show how MLP performs when new cell types exist in target datasets.

Our MLP classifier applies the SoftMax function as the final layer, which turns the values from the network prediction into probabilities for each cell type as an output. When the cell type exists in the reference, the probabilities of predicting cells belonging to this cell type should be much higher compared to probabilities of predicting cells belonging to other cell types. Therefore, when new cell types exist in the target, an ideal classifier should produce low probabilities for all cell types in the reference dataset. With this assumption, we conduct the following analyses to quantify and visualize the classification on new cell types.

We first remove one or two cell types from reference dataset, train the classifier and predict cells in the target dataset using MLP. We then extract out the maximum probability of each sample among all cell types. Although certain cells might be misclassified, we consider the maximum probability coming from the correct class in most cases. In the meantime, we have the ground truth of which cell type(s) are excluded, which can be presented as a binary vector. Therefore, an area under the receiver operating characteristic (AUROC) can be computed between the probability vector and the binary indicator. For the scenario where two cell types are excluded, we consider all missing cell types as one instead of drawing two AUROCs because we cannot know if there is one or more novel cell types in the target datasets beforehand. In total, we perform our analysis on all 29 experiments and show 4 of them as examples. All four results (Figure S11) show the new cell types are having relatively high AUC scores.

To visualize how cells are predicted when setting a threshold, we use 0.9 as cutoff to split cells into *assigned* and *unassigned* categories. We then visualize the *unassigned* cells to examine whether all of them belong to the excluded cell types. We use human PBMC batch effect and human pancreas dataset effect to illustrate scenarios when there exist sub-cell types and all major cell types respectively. In human PBMC batch effect analysis (Figure S12), when B cells are excluded from reference dataset, most of the *unassigned* cells belong to the original B cell cluster with a small fraction being wrongly labeled to T-sub cell types. However, when specifically investigating the original B cell cluster, we notice that almost half is assigned either to CD4 T+ cells or Dendritic cells. When CD14+ Monocytes are furthermore excluded, although most of *unassigned* cells lie in the original B cell and CD14+ Monocytes clusters, a large amount of them is assigned to T-sub types and NK cells. Also, half of CD14+ Monocytes are wrongly classified as FCRG3A+ Monocytes. In human pancreas dataset effect analysis (Figure S13), when acinar cells are excluded, the original ones are misclassified as ductal cells and *unassigned* cells become those cells on the border of each cell cluster. When alpha cells are excluded, although certain cells in the original alpha cell cluster are labeled as *unassigned*, most of them are classified as gamma cells.

**S5. Number of features has an impact on performance**

We also inspect how number of features might affect the prediction. We pick two experiments as illustrations. One experiment is using 8 samples from “Human PBMC lupus” batch2 under control status as reference to predict 8 IFN-β stimulated samples from the same batch. The other experiment is using one mouse in “Mouse brain FC” to predict another mouse “Mouse brain FC”. We set feature number from 100 to 5,000 with 100 as step size. For the first experiment, the performance reaches a peak around 500-600 features and decreases when feature number increases (Figure S15A). For the second experiment, the performance first increases and plateaus after 500 features (Figure S16A). The pattern can be fully explained by the tSNE dimension reduction plot (Figure S15B, S16B) for both experiments. When feature number increases, clusters first become tighter and then gradually over-clustered. For major cell types, over-clustering will not affect prediction, but for similar subtypes, it introduces biases. However, feature selection itself is a very interesting research topic in single-cell area. In our study, we choose 1,000 as number of features for further analysis because most experiments perform well.

**Supplementary Table 1: Mouse brain datasets used in this study**

|  | Dataset Description | Protocol | No. cells | No. major cell types (subtypes) |
| --- | --- | --- | --- | --- |
| Mouse brain FC  [2] | [GSE116470](https://www.ncbi.nlm.nih.gov/geo/query/acc.cgi?acc=GSE116470), Frontal cortex brain region, 7 male adult mice subjects | Drop-seq | 71,639 | 14 (81) |
| Mouse brain HC [2] | [GSE116470](https://www.ncbi.nlm.nih.gov/geo/query/acc.cgi?acc=GSE116470), Hippocampus cortex brain region, 6 male adult mice subjects |  | 53,204 | 12 (103) |
| Mouse brain pFC  [3] | [GSE124952](https://www.ncbi.nlm.nih.gov/geo/query/acc.cgi?acc=GSE124952), 6 saline-treated adult mice (2 in each 3 timepoints: control, 48h after cocaine withdrawal (CW), 15 days after CW) | 10X Chromium | 11,886 | 8 (9) |
| Mouse brain cortex  [4] | [SCP425](https://singlecell.broadinstitute.org/single_cell/study/SCP425/single-cell-comparison-cortex-data), cortex1 and cortex2 samples from one-month old mice | DroNc-seq | 1,452 (cortex1)  892 (cortex2) | 8 |
| Mouse brain Allen  [5] | [NeMO: dat-jb2f34y](https://portal.brain-map.org/atlases-and-data/rnaseq/mouse-whole-cortex-and-hippocampus-10x), 3 male adult mice with frontal cortex extracted | 10X Chromium | 65,944 | 8 (47) |

***Note**: We remove pericytes from Mouse brain cortex dataset. For Mouse brain Allen dataset, we extract out cells within ACA and PL;ILA;ORB brain regions and consider them as frontal cortex.

**Supplementary Table 2: Human PBMC Datasets used in this study**

|  | Dataset Description | Protocol | No. cells | No. major cell types (subtypes) |
| --- | --- | --- | --- | --- |
| Human PBMC lupus  [6] | [GSE96583](https://www.ncbi.nlm.nih.gov/geo/query/acc.cgi?acc=GSE96583), batch1, 8 SLE patients | 10X Chromium | 12,544 | 6 (8) |
|  | [GSE96583](https://www.ncbi.nlm.nih.gov/geo/query/acc.cgi?acc=GSE96583), batch2, 8 SLE patients untreated for 6 hours |  | 12,138 |  |
|  | [GSE96583](https://www.ncbi.nlm.nih.gov/geo/query/acc.cgi?acc=GSE96583), batch2, 8 SLE patients activated by IFN-β for 6 hours |  | 12,167 |  |
| Human PBMC protocols  [4] | [SCP424](https://singlecell.broadinstitute.org/single_cell/study/SCP424/single-cell-comparison-pbmc-data), pooled frozen 25million pbmc1 and within 4-hour fresh blood pbmc2 | Smart-seq2/ CEL-Seq2/  10X Chromium (v2) | 6,814 (pbmc1)  223  (pbmc2) | 6 (9) |
| Human PBMC FACS  [7] | [10X Genomics Datasets](https://www.10xgenomics.com/resources/datasets), fresh healthy Donor A with 10 bead-enriched subpopulations | FACS | 94,655 | 5 (10) |

***Note**: For Human PBMC 7 protocols dataset, we extract pbmc1 data with Smart-seq2, CEL-Seq2 and 10X Chromium protocols and pbmc2 data with Smart-seq2 data only.

**Supplementary Table 3: Human Pancreas datasets used in this study**

|  | Dataset Description | Protocol | No. cells | No. cell types |
| --- | --- | --- | --- | --- |
| Human Pancreas  [8] | [GSE85241](https://www.ncbi.nlm.nih.gov/geo/query/acc.cgi?acc=GSE85241), 4 dead donors (1 female, 3 males; variation in Age and BMI), 8 libraries | CEL-Seq2 | 2,018 | 6 |
| Human Pancreas  [9] | [E-MTAB-5061](https://www.ebi.ac.uk/arrayexpress/experiments/E-MTAB-5061/), 6 healthy and 4 T2D individuals (variation in healthy gender and age, BMI) | Smart-Seq2 | 2,038 | 6 |
| Human Pancreas [10] | [GSE81608](https://www.ncbi.nlm.nih.gov/geo/query/acc.cgi?acc=GSE81608), 12 Healthy and 6 T2D donors (balanced gender, varied age, BMI, weight) | SMARTer | 1,492 | 6 |

***Note:** We curate the human pancreas datasets only containing the 6 major cell types including alpha, beta, gamma, delta, acinar and ductal cells.

**Reference**

1. Selewa A, Dohn R, Eckart H, Lozano S, Xie B, Gauchat E, et al. Systematic comparison of high-throughput single-cell and single-nucleus transcriptomes during cardiomyocyte differentiation. Scientific reports. Nature Publishing Group; 2020;10:1–13.

2. Saunders A, Macosko EZ, Wysoker A, Goldman M, Krienen FM, Rivera H de, et al. Molecular Diversity and Specializations among the Cells of the Adult Mouse Brain. Cell. 2018;174:1015-1030.e16.

3. Bhattacherjee A, Djekidel MN, Chen R, Chen W, Tuesta LM, Zhang Y. Cell type-specific transcriptional programs in mouse prefrontal cortex during adolescence and addiction. Nature communications. Nature Publishing Group; 2019;10:1–18.

4. Ding J, Adiconis X, Simmons SK, Kowalczyk MS, Hession CC, Marjanovic ND, et al. Systematic comparison of single-cell and single-nucleus RNA-sequencing methods. Nature biotechnology. Nature Publishing Group; 2020;38:737–46.

5. Yao Z, van Velthoven CTJ, Nguyen TN, Goldy J, Sedeno-Cortes AE, Baftizadeh F, et al. A taxonomy of transcriptomic cell types across the isocortex and hippocampal formation. Cell. 2021;184:3222-3241.e26.

6. Kang HM, Subramaniam M, Targ S, Nguyen M, Maliskova L, McCarthy E, et al. Multiplexed droplet single-cell RNA-sequencing using natural genetic variation. Nature biotechnology. Nature Publishing Group; 2018;36:89.

7. Zheng GX, Terry JM, Belgrader P, Ryvkin P, Bent ZW, Wilson R, et al. Massively parallel digital transcriptional profiling of single cells. Nature communications. Nature Publishing Group; 2017;8:1–12.

8. Muraro MJ, Dharmadhikari G, Grün D, Groen N, Dielen T, Jansen E, et al. A single-cell transcriptome atlas of the human pancreas. Cell systems. Elsevier; 2016;3:385-394. e3.

9. Segerstolpe Å, Palasantza A, Eliasson P, Andersson E-M, Andréasson A-C, Sun X, et al. Single-cell transcriptome profiling of human pancreatic islets in health and type 2 diabetes. Cell metabolism. Elsevier; 2016;24:593–607.

10. Xin Y, Kim J, Okamoto H, Ni M, Wei Y, Adler C, et al. RNA sequencing of single human islet cells reveals type 2 diabetes genes. Cell metabolism. Elsevier; 2016;24:608–15.

Figure S1: Prediction ARI/macroF1 gains/losses with different reference size. The upper panel describes ARI, and the lower panel describes macroF1. (A)(E) 0 – 1,000 cells; (B)(F) 1,000 – 5,000 cells; (C)(G) 5,000 – 10,000 cells; (D)(H) 10,000+ cells. Similar to gains/losses in accuracy, scmap ranks top when the reference size is small. SVM related methods rank higher when number of cells increases. F-test on reference data ranks steadily as 1^st^ among all feature selection methods.

Figure S2: Prediction ARI/macroF1 gains/losses with different number of cell types in the reference dataset. (A)(C) <=10 cell types; (B)(D) > 10 cell types. scmap ranks higher when the number of cell types is smaller. SVM with RBF kernel ranks higher when number of cell types increases. F-test on reference data ranks steadily as 1^st^ among all feature selection methods.

Figure S3: Prediction performance gains/losses when using Human PBMC FACS data as target. (A) Accuracy; (B) ARI; (C) Macro F1. We observe the same pattern as the 29 experiments that MLP and SVM with different kernels are comparatively better than other prediction methods. In the meantime, F-test on reference dataset performs the best compared to others.

Figure S4: Prediction performance comparisons before and after imputation. (A) Accuracy; (B) ARI; (C) Macro F1. The imputation methods are performed on both reference and target datasets. The black, orange, blue, and green bars stand for MAGIC, SAVER, scVI and the one without imputation respectively.

Figure S5: Prediction performance comparisons before and after batch effect correction. (A) Accuracy; (B) ARI; (C) Macro F1. The batch effect correction is performed between reference and target datasets. The black, orange, and blue bars stand for Harmony, fastMNN and the one without batch effect correction respectively.

Figure S6: Impact of discrepancies between reference and target. (A) (B) describe the ARI and Macro F1 performance differences in mouse brain experiments with region effect and two dataset effects. (C) (D) describe the ARI and Macro F1 differences in human PBMC experiments with batch effect and clinical difference with sub-cell types, i.e., CD4+ T cells and CD8+ T cells. We take individual effect as baseline.

Figure S7: Impact of “pooling” on individual effect under intra-dataset and inter-dataset scenarios on ARI and Macro F1. (A)(B)(C) are under intra-dataset setting (black line indicates “pooling” all individuals) and (D) is under inter-dataset setting (black box indicates “pooling” all individuals).

Figure S8: Cell type annotations when combining “Mouse brain pFC” and “Mouse brain cortex” to predict the target from the “Mouse brain FC” dataset. (A) The blue dots in red box are cells from “Mouse brain cortex” and all other blue dots come from “Mouse brain pFC”. The orange dots are cells from “Mouse brain FC”. As shown in (B), corresponding cells in the red box contain a mixture of several cell types. (C) Ground truth cell types for target dataset. (D) Predicted cell types for target dataset. (D) shows some interneurons (green dots) misclassified as neurons (purple dots).

Figure S9: Prediction performance when using reference data with different sizes. The blue line, orange line and green line indicate Accuracy, ARI and macroF1 changes respectively. Results are based on 50 random shuffles when adding individuals (left panel) and cells (right panel). The shaded area is the 0.025 quantiles and 0.975 quantiles of the 50 results. (A) “Mouse brain FC” within dataset prediction using major cell types; (B) combines the “Mouse brain pFC” and “Mouse brain Allen” to predict one individual in “Mouse brain FC”; and (C) “Mouse brain FC” sub-cell types prediction. With more individuals being added, the performance increases and saturates in (A) and (B) but increases without saturation in (C).

Figure S10: Visualize removed cells from purifications. Orange dots are remained as reference dataset and blue dots are removed by different purification strategies. (A) “Human PBMC lupus”: use one lupus sample from batch1 to predict another sample from the same batch under the same condition. (B) “Mouse brain FC”: use one mouse subject to predict another mouse from the same dataset under the same condition on sub-cell types. As shown in the right panels, cells on boundaries of clusters are removed in probability-based purification.

Figure S11: ROCs for identifying novel cell types. The blue line is the ROC curve when one novel cell type exists, and the orange line describes the curve when there are two novel cell types. The excluded cell types are shown in figure legend. (A) Human PBMC lupus: use batch1 A to predict batch1 B; (B) Human pancreas: use one dataset to predict another dataset; (3) Mouse brain FC: uses one individual to predict another; (4) Mouse brain: one dataset to predict another dataset.

Figure S12: Prediction thresholding for MLP classifier when using Human PBMC lupus dataset batch1 A to predict batch1 B. The threshold is set at 0.9. (A) describes the *unassigned* cells when B cells are excluded and (B) describes the *unassigned* cells when B cells and CD14+ Monocytes are excluded.

Figure S13: Prediction thresholding for MLP classifier when using one Human pancreas dataset to predict another dataset. The threshold is set at 0.9. (A) describes the *unassigned* cells when acinar cells are excluded and (B) describes the *unassigned* cells when acinar cells and alpha cells are excluded.

Figure S14: Different cell type annotations between mouse brain datasets. The red box contains two lineages. The blue lineage is annotated as newly formed oligodendrocytes (NF Oligo) while the orange lineage is annotated as polydendrocytes.

Figure S15: Impact of feature number when using 8 samples from “Human PBMC lupus” batch2 to predict 8 IFN-β treated samples from the same batch. (A) shows Accuracy, ARI and Macro F1 performance changes when selecting 100 to 5,000 features and (B) shows tSNE visualizations when selecting 100, 500 and 2,000 features.

Figure S16: Impact of feature number when using one mouse subject from “Mouse brain FC” to predict another mouse subject from the same dataset under the same condition. (A) shows Accuracy, ARI and Macro F1 performance changes when selecting 100 to 5,000 features and (B) shows tSNE visualizations when selecting 100, 500 and 2,000 features.
