# Supplementary material for: Evaluation of some aspects in supervised cell type identification for single-cell RNA-seq: classifier, feature selection, and reference construction
Source: Genome Biol. 2021 Sep 9;22:264. doi: 10.1186/s13059-021-02480-2 (PMC8427961; doi:10.1186/s13059-021-02480-2)
Supplement: Supplementary file 4 — Additional file 4. Review history. [file 13059_2021_2480_MOESM4_ESM.docx]

Review History

**First round of review**

**Reviewer 1**

**Are you able to assess all statistics in the manuscript, including the appropriateness of statistical tests used?**

Yes, and I have assessed the statistics in my report.

**Comments to author:**

This manuscript presents a comprehensive evaluation of supervised cell type identification strategies. This is of broad interest to the single-cell community and the conclusions should be useful to the data analysis. There are certain points in the paper that may worth further considerations:

1. It is not directly clear from the manuscript what are used as the gold standard for cell types. Are these cell types inferred computationally or derived experimentally? For one dataset as an example (reference [32] Ding et al.), the original paper stated that 'We assigned each cluster a cell type identity based on known marker genes'. So it seems that the cell types are assigned computationally by other researchers using some methods. First, how reliable are these gold standards themselves? For example, will the choice of clusters in the original paper affect these gold standards? Second, will such computational gold standard biasedly favor certain kinds of methods that are more similar to the method used to derive the gold standard cell types in the first place? How about using datasets where cells are sorted experimentally, such as in https://www.nature.com/articles/ncomms14049 (data available at https://www.10xgenomics.com/resources/datasets Single Cell 3' Paper: Zheng et al. 2017 (v1 Chemistry))

2. Is it possible to benchmark the ability of different methods to identify new cell types, or cell types that are very different from any cell type in the reference?

3. Imputation methods for scRNA-seq has been a popular research topic. Is it possible to try imputation for the best performing method to see whether the performance can be further improved? Maybe just try the top methods listed in this benchmark paper https://genomebiology.biomedcentral.com/articles/10.1186/s13059-020-02132-x .

4. When pooling reference datasets, is there any procedure to remove the technical effect across different studies?

5. Consider using different colors to represent different methods in the Figure.

6. The GitHub page https://github.com/marvinquiet/Celltyping_RefConstruct cannot be accessed on the date of review (Jun 2). It seems that the correct URL is https://github.com/marvinquiet/RefConstruction_supervisedCelltyping?

**Reviewer 2**

**Are you able to assess all statistics in the manuscript, including the appropriateness of statistical tests used?**

Yes, and I have assessed the statistics in my report.

**Comments to author:**

Ma at al. presented an evaluation of 9 supervised cell type identification methods and assessed the impact of feature selection, prediction model and the choice of reference data and emphasized the importance of reference data that were used to train the supervised cell type identification methods.

Questions:

1. The title is a bit ambitious and confusing, does not precisely reflect the content of this manuscript

2. The authors assessed the effect of discrepancy between reference and target data on cell type identification accuracy. An assessment of the discrepancy or an illustration of the presence of discrepancy will be helpful. When pooling reference datasets, it was not clear whether the pooling was done by simple data concatenation or by batch effect correction (i.e. data integration). It will be helpful for readers to see if using the batch-corrected values as reference will improve cell type identification compared to non-corrected values.

3. It is not uncommon that target data have new or unknown cell types. For example, reference data have A, B, C while target data have B, C, D. Can supervised methods accurately classify D or force it to be one of A, B or C?

4. Authors concluded that pooling reference datasets will improve cell type identification of target data. However, this conclusion was drawn from only 2 datasets: one for mouse brain and the other human BPMC. In literature, many datasets derived from mouse brain or human PBMC have be published. Is it recommended to pool all available datasets or there is a saturation point? When more and more datasets are pooled, will more noise be also introduced and how this will affect the prediction accuracy? How do we select an optimal set of reference datasets to pool for classifying a specific target dataset?

5. Three quantitative metrics, Acc Gain/Loss, ARI Gain/Loss, macroF1 Gain/Loss, were used. I may have missed it somewhere, how was Gain/Loss calculated and what is the baseline? And among these three metrics, which one is more reliable? Starting from Figure 2, only Acc was used while the other two were dropped.

6. Authors highlighted the importance of reference data. Providing a guideline on how to assess if a reference dataset is comprehensive, reliable, has high quality, and have accurate cell type annotation will be very useful to readers to choose the best reference for their target data.

7. Authors used 1000 features to assess the impact of feature selection methods. Does the number of features (500, 1000, 2000, 3000, etc) also have an impact on the performance?

8. Is Figure 1 generated using all datasets and does it include all predictions?

9. How do F-test and FEAST select relevant features from reference data? It is performed using scanpy package. What is the underlying methodologist of FEAST and F-test?

10. MLP is recommended as the best method. Usually artificial neural network need large sample size to train the network. Does the MLP method perform well on small reference data? And how many layers and nodes are in the MLP network?

11. Line 82, there should be more existing methods.

Name	Language	Underlying classifier	Reference	Journal

ACTINN	Python	Neural network	Ma F et al, 2019	Bioinformatics

AltAnalyze	?

Capybara	R	Quadratic programming	Kong W et al, 2020	BioRxiv

CaSTLe	R	Random forest	Lieberman Y et al, 2018	PLOS ONE

CellAssign	R	Probabilistic Bayesian model	Zhang AW et al, 2019	Nat Methods.

Cell-BLAST	Python	Cell-to-cell similarity	Cao ZJ et al, 2019	Nature Communications

CellFishing	Julia	approximate k-NN (locality sensitive hashing + hamming distance)	Genome Biology

CellO	Python	hierarchical prediction against the Cell Ontology	Bernstein MN et al, 2021

CHETAH	R	Correlation to training set	Kanter JK et al, 2019	Nucleic Acids Research

CIPR	R, Shinyapp	Dot product, Spearman, Pearson	Ekiz HA et al, 2020	BMC Bioinformatics

ClustifyR	R	Spearman, Pearson, Kendall, cosine	Fu R et al, 2020	F1000Res.

DigitalCellSorter	Python	Voting based on cell type markers	Domanskyi S et al, 2019

Garnett	R	Generalized linear model	Pliner et al, 2019	Nat Methods.

kNN	Python	kNN (k = 9)	Pedregosa F et al, 2011

LAmbDA	Python	Random forest	Johnson TS et al, 2019	Bioinformatics

LDA	Python	LDA	Pedregosa F et al, 2011

MARS	Python	Supervised, autoencoder neural network	Nat Methods.

Moana	Python	SVM with linear kernel	Wagner et al, 2018	BioRxiv

NMC	Python	NMC	Pedregosa F et al, 2011

northstar	Python	Graph based(?) data merging	Scientific Reports

OnClass	Python	kNN and Bilinear Neural Network	BioRxiv

Polled Digital Cell Sorter	Python	Marker based voting system	BMC Bioinformatics

RaceID	R	Based on transcript counts obtained with unique molecular identifies	Dominic G et al, 2015

RF	Python	RF (50 trees)	Pedregosa F et al, 2011

scANVI	Python	kNN classifier	Xu C et al, 2021	Mol Syst Biol

scCATCH	R	Scoring system	Shao X et al, 2020	iScience

scClassifR	R	RBF SVM-based machine learning	BioRxiv

scClassify	R, Shinyapp	Weighted kNN classifier	Lin Y et al, 2019	Mol Syst Biol

scHCL	R	Pearson correlation (for human)	Nature

SciBet	web client	Supervised(?), gene expression probability of reference, Maximum likelihood based? Nature Communications

scID	R	LDA	Boufea K et al	iScience

SCINA	R	Bimodal distribution fitting for marker genes	Zhang Z et al, 2019	Genes

scLearn	Discriminant Correlation Anaysis based matrix decompositon	Science Advances

scmapcell	R	kNN	Kiselev VY et al, 2018	Nat Methods.

scmapcluster	R	Nearest median classifier	Kiselev VY et al, 2018	Nat Methods.

scMatch	Python	Spearman, Pearson	Hou R et al, 2019	Bioinformatics

scMCA	R	Pearson correlation (for mouse)	Methods Mol Biol

scNym	Python	Adversarial Neural Network	Kimmel JC et al, 2020	Genome Research

scPred	R	SVM with radial kernel	Alquicira HJ et al, 2018	Genome Biology

SCSA	Python	Scoring system	Cao Y et al, 2020	Frontiers in Genetics

scVI	Python	Neural network	Lopez R et al, 2018

scVI/scANVI	Python	Mol Syst Biol

Seurat	R	Nature Biotechnology

SINCERA	R	Gou M et al, 2015

SingleCellNet	R	Supervised, Random forest	Tan Y et al, 2018	Cell Systems

SingleR	R	Correlation to training set	Aran D et al, 2019	Nature immunology

SNN-Cliq	Python	Xu C and Su Z, 2015

superCT	Web app	Artificial Neural Network	Xie P et al, 2019	Nucleic Acids Research

SVM	Python	SVM (linear kernel)	Pedregosa F et al, 2011

SVMrejection	Python	SVM (linear kernel)	Pedregosa F et al, 2011

**Authors Response**

**Point-by-point responses to the reviewers’ comments:**

Response to Reviewer 1’s comments:

This manuscript presents a comprehensive evaluation of supervised cell type identification strategies. This is of broad interest to the single-cell community and the conclusions should be useful to the data analysis.

*We thank the reviewer for the positive comments.*

There are certain points in the paper that may worth further considerations:

(Comment 1) It is not directly clear from the manuscript what are used as the gold standard for cell types. Are these cell types inferred computationally or derived experimentally? For one dataset as an example (reference [32] Ding et al.), the original paper stated that 'We assigned each cluster a cell type identity based on known marker genes'. So it seems that the cell types are assigned computationally by other researchers using some methods. First, how reliable are these gold standards themselves? For example, will the choice of clusters in the original paper affect these gold standards? Second, will such computational gold standard biasedly favor certain kinds of methods that are more similar to the method used to derive the gold standard cell types in the first place? How about using datasets where cells are sorted experimentally, such as in https://www.nature.com/articles/ncomms14049 (data available at https://www.10xgenomics.com/resources/datasets Single Cell 3' Paper: Zheng et al. 2017 (v1 Chemistry))

*This is a great comment. We used the cell type labels annotated by the original papers and taken them as “gold standards”. We confirm that all of them are acquired based on unsupervised cell clustering followed by computational or manual annotation using known marker genes. We completely agree that these data can only be taken as “silver standard”.*

*The main reason for such choice is the lack of real “gold” standard dataset, since only a handful of datasets with cell types identified by experimental cell sorting are available. There are also problems with experimental cell sorting data. Since the cell surface markers are not available for all cell types, these data are often biased toward certain cell types. Thus, these data cannot be used as reference, and can only be used as target in method evaluation. For computationally derived cell types, although the cell types are dependent on the clustering analysis, manual efforts have been made in the literatures to select the best number of clusters and combine or separate certain clusters, thus the results should be mostly accurate. There sure will be mislabels in these data, but we believe those are random errors and shouldn’t have systematic impact on the methods being evaluated. For the comment about computational gold standard biasedly favor certain kinds of methods that are more similar to the method used to derive the gold standard cell types: we benchmark supervised methods with the gold standards generated by unsupervised methods. It’s unlikely that certain supervised methods are more “similar” to an unsupervised method. Taken these together, even though the gold standards used in this work are not 100% accurate, we believe the general conclusion of our work is valid.*

*This being said, we performed additional analyses using the FACS-sorted human PBMC (Zheng et al. 2017) as target and conduct 4 experiments including: cross-validation using original cell types, cross-validation using curated major cell types (B cells, NK cells, CD4+ T cells, CD8+ T cells and Monocytes), use one individual from human PBMC lupus (Kang et al. 2018) to predict PBMC FACS and use fresh sample sequenced by 10X from Human PBMC protocols (Ding et al. 2020) to predict PBMC FACS. The results from the four experiments are summarized in Additional file 1: Fig S3 (also shown below).*

*As shown in the figure, MLP and SVM are still among the best performers, and F-test on reference dataset generates the overall best prediction results. Therefore, our original conclusion that MLP with F-test feature selection is the best overall performer still holds no matter using computationally derived cell types or FACS-sorted cell types as target. To add on, this FACS data does not contain comprehensive cell types. For example, CD16+ Monocytes, Megakaryocytes and Dendritic cell disappear from the Zheng et al. but all exist in two other PBMC datasets with computationally derived annotations.*

*We have added a new subsection Impact of cell type annotations in Results section to discuss this point. The details of the experimental design can be found in Additional file 1: Section S2 under Datasets for comparing cell type annotations.*

*Reference for Comment 1:*

*Zheng, Grace XY, et al. "Massively parallel digital transcriptional profiling of single cells." Nature communications 8.1 (2017): 1-12.*

*Kang, Hyun Min, et al. "Multiplexed droplet single-cell RNA-sequencing using natural genetic variation." Nature biotechnology 36.1 (2018): 89-94.*

*Ding, Jiarui, et al. "Systematic comparison of single-cell and single-nucleus RNA-sequencing methods." Nature biotechnology 38.6 (2020): 737-746.*

(Comment 2) Is it possible to benchmark the ability of different methods to identify new cell types, or cell types that are very different from any cell type in the reference?

*In general, the supervised methods can only predict class labels included in the reference data. Thus, they cannot directly identify new cell types. Cell types that are very different from any cell type in the reference can be considered as “new” cell types. Without additional procedure, any new cell type in the target data will be forced into one of the existing cell types. In contrast, unsupervised and semi-supervised methods are better at dealing with unseen labels. We actually provided some discussions on this issue in the last paragraph of the Discussion section.*

*This being said, it is possible to set a threshold on the prediction probabilities to classify some cells as “unassigned” (this is the strategy used in scmap and CHETAH). The new cell types can hopefully be classified as unassigned for further analyses to identify new cell type. We performed additional analyses to investigate such a strategy on MLP. We first randomly remove one or two cell types from reference dataset (thus there are new cell types in the target data), and then do prediction. Intuitively, if a cell belongs to a new cell type, its maximum prediction probabilities for existing cell types should be low, i.e., the prediction has higher entropy because the cell is not similar to any of the reference cell type. We then evaluate whether such maximum prediction probabilities are related to the new cell type using receiver operating characteristic (ROC) curves, where the maximum prediction probabilities are deemed scores for calling unassigned, and the outcome is the status of new cell type. Below, we show the results from four datasets (the figure is now included in Additional file 1: Fig S11). It demonstrates that such an approach can reasonably call new cell types as unassigned. The performance of such procedure highly depends on the data. When the new cell type is very different from the existing ones (the mouse brain results in Fig S11C, S11D), the results can be very good. Otherwise (human pancreas data in Fig S11B), the results can be rather poorer.*

*Of course, calling unassigned cell type is not equal to identifying new cell type, but it’s the first step. How to use supervised method to identify new cell types is certainly an interesting and important question and is our research topic in the near future.*

*We have added a section named Predicting novel cell types under Results section to discuss this point and analyses details can be found in Additional file 1: Section S4.*

(Comment 3) Imputation methods for scRNA-seq has been a popular research topic. Is it possible to try imputation for the best performing method to see whether the performance can be further improved? Maybe just try the top methods listed in this benchmark paper https://genomebiology.biomedcentral.com/articles/10.1186/s13059-020-02132-x.

*Thank you for your suggestions. We select three imputation methods: MAGIC (smoothing-based method), SAVER (model-based) and scVI (data reconstruction based on deep-learning), then perform them on both reference and target datasets on all our 29 experiments. Since we have observed that MLP with F-test as feature selection generates overall best results, we perform imputations on this combination and compare to our original results. Results are summarized in Additional file 1: Fig S4 (the accuracy comparison is shown below).*

*As shown in our results, there is no specific imputation method outperforming others or the original prediction results. Therefore, imputation may not be a necessary step for cell type classification. We have added a new subsection Impact of data preprocessing under the Results section. The implementation details are provided in Methods section under Implementation of imputation.*

(Comment 4) When pooling reference datasets, is there any procedure to remove the technical effect across different studies?

*In our previous version, we directly concatenate the reference datasets without removing the technical effects. Because most of our pooling experiments happen within a dataset and individual effects are quite small in our experiments (shown in Figure 4 and our analysis can be found in Results section under Condition effect), we do not remove technical effects among individuals.*

*This question inspires us to correct the batch effect between reference and target datasets and examine if performance becomes any better. We select two batch effect removal methods (Harmony by Korsunsky et al. 2019 and fastMNN by Haghverdi et al. 2018, Lun 2019) to eliminate the discrepancies between reference and target datasets. We then compare the prediction performances to our original results. The results are summarized in Additional file: Fig S5 (the accuracy is shown below). We believe batch effect removal does not improve the prediction performance in most cases (also when across different studies, e.g., Muraro to Seg, Seg Xin to Muraro, Seg to Muraro). More discussions can be found in Results section under Impact of data preprocessing. The implementation details are provided in Methods section under Implementation of batch effect correction.*

*Reference for Comment 4:*

*Korsunsky, Ilya, et al. "Fast, sensitive and accurate integration of single-cell data with Harmony." Nature methods 16.12 (2019): 1289-1296.*

*Haghverdi, Laleh, et al. "Batch effects in single-cell RNA-sequencing data are corrected by matching mutual nearest neighbors." Nature biotechnology 36.5 (2018): 421-427.*

*Lun, A. "Further MNN algorithm development." (2019).*

(Comment 5) Consider using different colors to represent different methods in the Figure.

*Thank you for the suggestions. We have changed the color for different methods in Figure1, Figure2, Figure 3 and Figure 6. For the newly added figures, we also apply the same color scheme.*

(Comment 6) The GitHub page https://github.com/marvinquiet/Celltyping_RefConstruct cannot be accessed on the date of review (Jun 2). It seems that the correct URL is https://github.com/marvinquiet/RefConstruction_supervisedCelltyping?

*Thank you for pointing that out. We have updated the URL in both Abstract and Availability of data and materials.*

Response to Reviewer 2’s comments:

Ma at al. presented an evaluation of 9 supervised cell type identification methods and assessed the impact of feature selection, prediction model and the choice of reference data and emphasized the importance of reference data that were used to train the supervised cell type identification methods.

Questions:

(Comment 1) The title is a bit ambitious and confusing, does not precisely reflect the content of this manuscript

*Thank you for the comment. We have changed the title to “Evaluation of some aspects in supervised cell type identification for single-cell RNA-seq: classifier, feature selection, and reference construction”.*

(Comment 2) The authors assessed the effect of discrepancy between reference and target data on cell type identification accuracy. An assessment of the discrepancy or an illustration of the presence of discrepancy will be helpful. When pooling reference datasets, it was not clear whether the pooling was done by simple data concatenation or by batch effect correction (i.e. data integration). It will be helpful for readers to see if using the batch-corrected values as reference will improve cell type identification compared to non-corrected values.

*Thank you for your comment. In our previous analysis, we have simply concatenated datasets together and classified the cell types. In the revised manuscript, we evaluate whether batch effect removal can improve the prediction results. We include two batch effect removal methods: Harmony (Korsunsky et al. 2019) and fastMNN (Haghverdi et al. 2018; Lun 2019). We remove the batch effects between the reference and target dataset and perform on all 29 experiments with the combination of F-test and MLP. The results are summarized in Additional file: Fig S5 (the accuracy is also shown below).*

*As shown in the figures, the performances with and without batch effect removal are comparable. In fact, in most cases the prediction performances without batch effect correction are slightly higher. Thus, we believe batch effect removal is not necessary in supervised cell identification. We have discussed this point in Results section under Impact of data preprocessing, and the implementation details are added in Methods section under Implementation of batch effect correction.*

*Reference for Comment 2:*

*Korsunsky, Ilya, et al. "Fast, sensitive and accurate integration of single-cell data with Harmony." Nature methods 16.12 (2019): 1289-1296.*

*Haghverdi, Laleh, et al. "Batch effects in single-cell RNA-sequencing data are corrected by matching mutual nearest neighbors." Nature biotechnology 36.5 (2018): 421-427.*

*Lun, A. "Further MNN algorithm development." (2019).*

(Comment 3) It is not uncommon that target data have new or unknown cell types. For example, reference data have A, B, C while target data have B, C, D. Can supervised methods accurately classify D or force it to be one of A, B or C?

*In general, the supervised methods can only predict class labels included in the reference data. Thus, they cannot directly identify new cell types. Cell types that are very different from any cell type in the reference can be considered as “new” cell types. Without additional procedure, any new cell type in the target data will be forced into one of the existing cell types. In contrast, unsupervised and semi-supervised methods are better at dealing with unseen labels. We actually provided some discussions on this issue in the last paragraph of the Discussion section.*

*This being said, it is possible to set a threshold on the prediction probabilities to classify some cells as unassigned (this is the strategy used in scmap and CHETAH). The new cell types can hopefully be classified as unassigned for further analyses to identify new cell type. We performed additional analyses to investigate such a strategy on MLP. We first randomly remove one or two cell types from reference dataset (thus there are new cell types in the target data), and then do prediction. Intuitively, if a cell belongs to a new cell type, its maximum prediction probabilities for existing cell types should be low, i.e., the prediction has higher entropy because the cell is not similar to any of the reference cell type. We then evaluate whether such maximum prediction probabilities are related to the new cell type using receiver operating characteristic (ROC) curves, where the maximum prediction probabilities are deemed scores for calling “unassigned”, and the outcome is the status of new cell type. Below, we show the results from four datasets (the figure is now included in Additional file 1: Fig S11). It demonstrates that such an approach can reasonably call new cell types as unassigned. The performance of such procedure highly depends on the data. When the new cell type is very different from the existing ones (the mouse brain results in Fig S11C, S11D), the results can be very good. Otherwise (human pancreas data in Fig S11B), the results can be rather poorer.*

*Of course, calling unassigned cell type is not equal to identifying new cell type, but it’s the first step. How to use supervised method to identify new cell types is certainly an interesting and important question and is our research topic in the near future.*

*We have added a section named Predicting novel cell types under Results section to further discuss our results and analyses details can be found in Additional file 1: Section S4.*

(Comment 4) Authors concluded that pooling reference datasets will improve cell type identification of target data. However, this conclusion was drawn from only 2 datasets: one for mouse brain and the other human BPMC. In literature, many datasets derived from mouse brain or human PBMC have be published. Is it recommended to pool all available datasets or there is a saturation point? When more and more datasets are pooled, will more noise be also introduced and how this will affect the prediction accuracy? How do we select an optimal set of reference datasets to pool for classifying a specific target dataset?

*This is a great point. To examine whether the saturation point exists, we conduct three additional analyses. We use the mouse brain datasets because they contain more individuals and cells compared to other datasets, which allows the exploration of performance saturation. We performed three types of analyses: (1) major cell type prediction within dataset; (2) major cell type prediction across different datasets; (3) sub-cell type prediction within dataset. We first use cells from different number of individuals as reference to examine whether the performance will saturate. Furthermore, we pool the cells from all individuals and randomly sample different numbers of cells as reference. This averages out the individual effects, and only investigate whether there will be saturation using more cells in the reference. More details of the analyses can be found in Additional file 1: Section S2 under Datasets for pooling saturation analysis and Section S3 under Analyses details on pooling saturation. The results are summarized in Additional file 1: Fig S9 (also shown below).*

*As shown in the figures, using larger reference indeed could lead to performance saturation overall. This is especially clear when predicting major cell types (Figure A, and left panel of Figure B). For the sub-cell type prediction (Figure C), using larger reference leads to better performance. However, we do not observe a clear saturation, and we believe the performance can be further improved with larger reference. This makes sense because sub-cell types are more similar, so the signal-to-noise ratio in the data is much lower compared to the major cell types. In order to observe saturation in these analyses, we will need larger reference.*

*Another finding from this analysis is that pooling individuals can potentially lead to faster saturation. This is very pronounced in sub-cell type prediction (Figure B). The right panel shows that the performance is saturated from the start (3000 cells) when cells are sampled from a pool of individuals. When adding each individual at a time (left panel), it requires 4 individuals (around 40,000 cells) to reach saturation. These results are consistent with our findings that “pooling” individuals can achieve better prediction performance.*

*For the question about larger reference data may introduce more noise: we do observe that including more cells in the reference could sometimes results in worse performance (Figure A and C right panels). However, the performance will increase back when the reference data become even larger. Regarding how to assess and select an optimal set of reference: this is related to the comment 6 below. We consider this a stand-alone project which involves extensive data analysis, method development and evaluation.*

*We have added related paragraphs in the Results section under Pooling references improves the prediction results to discuss more about these observations and analyses details are provided in Additional file 1: Section S3.*

(Comment 5) Three quantitative metrics, Acc Gain/Loss, ARI Gain/Loss, macroF1 Gain/Loss, were used. I may have missed it somewhere, how was Gain/Loss calculated and what is the baseline? And among these three metrics, which one is more reliable? Starting from Figure 2, only Acc was used while the other two were dropped.

*The calculation is demonstrated in the Additional file: S1 section. Briefly, we apply 9 classifiers and 6 feature selection methods, and the results for each dataset are arranged into a 9x6 performance table. We calculate the overall mean of each table as the baseline for each dataset, and then subtract the mean from each table to remove the dataset effect. Next, we compute the row and column averages for the normalized table to obtain the marginal performance for each classifier and feature selection method. Such marginal performances are the gains or losses relative to the baseline performance. Finally, we sort the tables by rows and columns with the top left element has the most performance gain and bottom right element has the most performance loss.*

*The three metrics are describing different perspectives of the prediction in which accuracy focuses on an overall classification regardless of imbalanced labels, ARI focuses on the cluster assignment without considering the labels of the clusters, MacroF1 represents an average of precision and recall regarding the label distribution, which is perhaps more meaningful when the cluster sizes are very unbalanced. All of them are reliable metrics but focusing on different perspectives. The reason why we only show Accuracy is because it has the most straightforward meaning and easy to understand. We have observed the same pattern among the three metrics in all analyses, as shown in Figure 1. Therefore, for simplification, we just show Accuracy for the rest of the figures. We have now included the ARI and Macro F1 performances for testing the impact of the reference data size and the impact of number of cell types in Additional file 1: Fig S1, Fig S2.*

(Comment 6) Authors highlighted the importance of reference data. Providing a guideline on how to assess if a reference dataset is comprehensive, reliable, has high quality, and have accurate cell type annotation will be very useful to readers to choose the best reference for their target data.

*This is a great question. As shown in saturation analysis (Additional file 1: Fig S9C), even though using larger reference dataset can improve results in general, adding certain individual can indeed result in worse performance in some cases. Therefore, how to assess and select reference dataset is a very interesting and important question. We consider this a stand-alone topic that requires extensive method development and evaluation, which is beyond the scope of this work. But we will certainly explore it in our future research.*

*At this point, we suggest pooling all datasets together because “pooling” averages out individual effects and provides larger reference data, which generate the best prediction performance in most cases.*

*We have added a few sentences to discuss this topic in Discussion section.*

(Comment 7) Authors used 1000 features to assess the impact of feature selection methods. Does the number of features (500, 1000, 2000, 3000, etc) also have an impact on the performance?

*Yes, we have tried different number of features in two experiments (Additional file 1: Section S5) and the results are summarized in Additional file 1: Fig S15, Fig S16. The number of features selected is dependent on different experiments. As shown in Fig S15, the performance first increases with more features and then deteriorates. But in Fig S16, the performance first increases and then plateaus. The number of features selected can be dynamically decided by running experiments multiple times but can consume a lot of time, therefore, we consider choosing 1,000 can generate descent prediction results most of the times.*

(Comment 8) Is Figure 1 generated using all datasets and does it include all predictions?

*Yes, it includes using all datasets and all predictions.*

(Comment 9) How do F-test and FEAST select relevant features from reference data? It is performed using scanpy package. What is the underlying methodologist of FEAST and F-test?

*F-test and FEAST are both performed using the FEAST (https://github.com/suke18/FEAST) package with one having cell type annotations as input and the other one with number of cell types as input parameter. The methods are detailed in FEAST paper (Su et al., 2021).*

*Briefly speaking, FEAST is an unsupervised feature selection method. It utilizes an underlying consensus clustering to derive cell clusters and then use F-test to calculate feature significance. Then, genes are ranked by F-statistics and the top can be selected as FEAST features. As for F-test, since the cell types are known in reference dataset, one can directly apply F-test to calculate feature significance.*

*We have added some language to briefly introduce these procedures in the Results Section under Study design: Feature selection methods.*

*Reference for Comment 9:*

*Su, Kenong, Tianwei Yu, and Hao Wu. "Accurate feature selection improves single-cell RNA-seq cell clustering." Briefings in Bioinformatics (2021).*

(Comment 10) MLP is recommended as the best method. Usually artificial neural network need large sample size to train the network. Does the MLP method perform well on small reference data? And how many layers and nodes are in the MLP network?

*This is a very good question. We also take this into consideration and split our experiments by number of cells into four categories: (1) 0 - 1,000 cells; (2) 1,000 – 5,000 cells; (3) 5,000 – 10,000 cells; and (4) over 10,000 cells. The results are summarized in Figure 2 (also shown below). As shown in the figure, when the sample size is relatively small, scmap performs comparatively better among all classifiers. When the sample size increases, MLP along with SVM methods rank higher. It does not mean other methods become worse with more cells, but rather because the supervised machine learning and deep learning methods become better for larger reference datasets. As for ItClust and MARS, which are two semi-supervised methods designed for detecting novel cell types, do not perform better than other classifiers in the celltyping tasks.*

*For your second question, the number of layers and nodes depends on the sample size. When the sample size is less than 5,000, we make the MLP with 2 hidden layers with nodes number as [64, 16] and when it is over 5,000, we enlarge it to 5 hidden layers with nodes number as [128, 64, 32, 16, 8] respectively.*

(Comment 11) Line 82, there should be more existing methods.

Name	Language	Underlying classifier	Reference	Journal

ACTINN Python Neural network Ma F et al, 2019	Bioinformatics

AltAnalyze	?

Capybara	R	Quadratic programming	Kong W et al, 2020	BioRxiv

CaSTLe R	Random forest	Lieberman Y et al, 2018 PLOS ONE

CellAssign	R	Probabilistic Bayesian model	Zhang AW et al, 2019	Nat Methods.

Cell-BLAST	Python Cell-to-cell similarity Cao ZJ et al, 2019	Nature Communications

CellFishing	Julia	approximate k-NN (locality sensitive hashing + hamming distance)	Genome Biology

CellO	Python hierarchical prediction against the Cell Ontology	Bernstein MN et al, 2021

CHETAH R	Correlation to training set	Kanter JK et al, 2019	Nucleic Acids Research

CIPR	R, Shinyapp	Dot product, Spearman, Pearson Ekiz HA et al, 2020	BMC Bioinformatics

ClustifyR	R	Spearman, Pearson, Kendall, cosine	Fu R et al, 2020	F1000Res.

DigitalCellSorter	Python Voting based on cell type markers	Domanskyi S et al, 2019

Garnett R	Generalized linear model	Pliner et al, 2019	Nat Methods.

kNN	Python kNN (k = 9)	Pedregosa F et al, 2011

LAmbDA Python Random forest	Johnson TS et al, 2019 Bioinformatics

LDA	Python LDA	Pedregosa F et al, 2011

MARS	Python Supervised, autoencoder neural network	Nat Methods.

Moana	Python SVM with linear kernel Wagner et al, 2018	BioRxiv

NMC	Python NMC	Pedregosa F et al, 2011

northstar	Python Graph based(?) data merging	Scientific Reports

OnClass Python kNN and Bilinear Neural Network	BioRxiv

Polled Digital Cell Sorter	Python Marker based voting system	BMC Bioinformatics

RaceID R	Based on transcript counts obtained with unique molecular identifies	Dominic G et al, 2015

RF	Python RF (50 trees)	Pedregosa F et al, 2011

scANVI Python kNN classifier Xu C et al, 2021	Mol Syst Biol

scCATCH R	Scoring system Shao X et al, 2020	iScience

scClassifR	R	RBF SVM-based machine learning	BioRxiv

scClassify	R, Shinyapp	Weighted kNN classifier Lin Y et al, 2019	Mol Syst Biol

scHCL	R	Pearson correlation (for human)	Nature

SciBet web client Supervised(?), gene expression probability of reference, Maximum likelihood based? Nature Communications

scID	R	LDA	Boufea K et al iScience

SCINA	R	Bimodal distribution fitting for marker genes	Zhang Z et al, 2019	Genes

scLearn	Discriminant Correlation Anaysis based matrix decompositon	Science Advances

scmapcell	R	kNN	Kiselev VY et al, 2018 Nat Methods.

scmapcluster	R	Nearest median classifier	Kiselev VY et al, 2018 Nat Methods.

scMatch Python Spearman, Pearson	Hou R et al, 2019	Bioinformatics

scMCA	R	Pearson correlation (for mouse)	Methods Mol Biol

scNym	Python Adversarial Neural Network	Kimmel JC et al, 2020	Genome Research

scPred R	SVM with radial kernel Alquicira HJ et al, 2018	Genome Biology

SCSA	Python Scoring system Cao Y et al, 2020	Frontiers in Genetics

scVI	Python Neural network Lopez R et al, 2018

scVI/scANVI	Python	Mol Syst Biol

Seurat R	Nature Biotechnology

SINCERA R	Gou M et al, 2015

SingleCellNet	R	Supervised, Random forest	Tan Y et al, 2018	Cell Systems

SingleR R	Correlation to training set	Aran D et al, 2019	Nature immunology

SNN-Cliq	Python	Xu C and Su Z, 2015

superCT Web app Artificial Neural Network	Xie P et al, 2019	Nucleic Acids Research

SVM	Python SVM (linear kernel)	Pedregosa F et al, 2011

SVMrejection	Python SVM (linear kernel)	Pedregosa F et al, 2011

*We appreciate the reviewer for providing such a comprehensive reference list. We cited some of the most popular methods in the Background section (e.g., scPred, Garnett, CellAssign, SingleCellNet). There are a large number of methods developed for this topic and a lot of them have been benchmarked in Abdelaal et al. In this work, we select a few top performers in Abdelaal et al. with some additions that are not included in the Abdelaal et al paper. Our major focus is to include a few methods in each of the three categories: (1) the traditional supervised machine learning and deep learning methods; (2) correlation-based methods; and (3) semi-supervised deep learning methods.*

*Reference for Comment 11:*

*Alquicira-Hernandez, Jose, et al. "scPred: accurate supervised method for cell-type classification from single-cell RNA-seq data." Genome biology 20.1 (2019): 1-17.*

*Pliner, Hannah A., Jay Shendure, and Cole Trapnell. "Supervised classification enables rapid annotation of cell atlases." Nature methods 16.10 (2019): 983-986.*

*Zhang, Allen W., et al. "Probabilistic cell-type assignment of single-cell RNA-seq for tumor microenvironment profiling." Nature methods 16.10 (2019): 1007-1015.*

*Tan, Yuqi, and Patrick Cahan. "SingleCellNet: a computational tool to classify single cell RNA-Seq data across platforms and across species." Cell systems 9.2 (2019): 207-213.*

*Abdelaal, Tamim, et al. "A comparison of automatic cell identification methods for single-cell RNA sequencing data." Genome biology 20.1 (2019): 1-19.*

**Second round of review**

**Reviewer 1**

The authors have addressed all my concerns.

**Reviewer 2**

The authors have addressed all the comments and revised the manuscript accordingly. I don't have further questions.

Many thanks to the authors for addressing all the comments and revising the manuscript accordingly. In the past few years, more than 50 algorithms have been developed for automated cell type identification. Several benchmark studies have also been published. In this field, it has been well accepted that the reference is far more important than the algorithm itself. Simple methods such as svm, correlation or regression are able to predict cell types accurately as long as an accurate and relevant reference is provided. However, it is challenging to build high-standard reference. As such, with all these available methods and benchmarks, manual cell type annotation is still the most commonly adopted method instead of using automated methods, due to the lack of "gold-standard" reference. Robust cell type annotation still remains a major challenge in single-cell transcriptomics, especially when handling very large datasets. How to evaluate or build a good reference for automated cell type annotation has been a burning question to many searchers in this field. Building such reference is currently one of most urgent tasks. Recently, several labs have built single-cell atlases, which are in turn used as references for their automated cell type annotations. As expected, predictions based on different references built by different labs give inconsistent annotations. How we compare, benchmark, combine and synergize these references is one of the more pressing issues that need to be addressed.
